# Investigating the effects of circadian rhythm on the human skin lipidome

**DOI:** 10.1039/d5an00665a

**Published:** 2025-10-14

**Authors:** Caroline Géhin, Amanda V. Witter, Lu Wang, Perdita E. Barran, Stephen J. Fowler, Drupad K. Trivedi

**Affiliations:** a Michael Barber Centre for Collaborative Mass Spectrometry, Manchester Institute of Biotechnology, Department of Chemistry, University of Manchester Princess Street Manchester M1 7DN UK drupad.trivedi@manchester.ac.uk; b Faculty of Biology, Medicine and Health, School of Biological Sciences, University of Manchester Manchester UK; c NIHR Manchester Biomedical Research Centre, Manchester University Hospitals NHS Foundation Trust Manchester UK

## Abstract

The circadian rhythm is a 24 h cycle that harmonises the activity of organs – including the skin – to a daily routine using neurological and hormonal signals. Limited research has been done to understand the effects of the circadian rhythm on the skin lipidome. We used reversed-phase liquid chromatography-mass spectrometry (RPLC-MS) in a longitudinal study to investigate temporal changes to the skin lipidome over a 24 h cycle for eight healthy participants. All statistical analyses were performed with a group-mean and individual-mean data approach. Using cosinor analysis *p*-values, a total of 29 metabolites (0.67% of all detected metabolites) exhibited a statistically significant circadian rhythmicity across participants; however, individually, a range of 3.51–18.53% of metabolites were considered rhythmic. The use of FDR *q*-values and Lomb–Scargle analysis showed no circadian metabolites. Using PCA and PLS-DA, no significant clustering based on timepoints was observed across participants; however, half of individuals showed significant metabolite clustering at 07:30. Further, sebum-specific squalene and sapienic acid as well as stratum corneum-specific cholesterol sulfate showed no significant differences in concentrations across timepoints. While individuals exhibited temporal differences, as an averaged healthy cohort the impacts by the circadian rhythm or time of sampling were considered negligible.

## Abstract 2

The circadian rhythm is a 24 h cycle that harmonises the activity of organs – including the skin – to a daily routine using neurological and hormonal signals. We show that majority of sebum does not show this rhythm.

## Graphical abstract

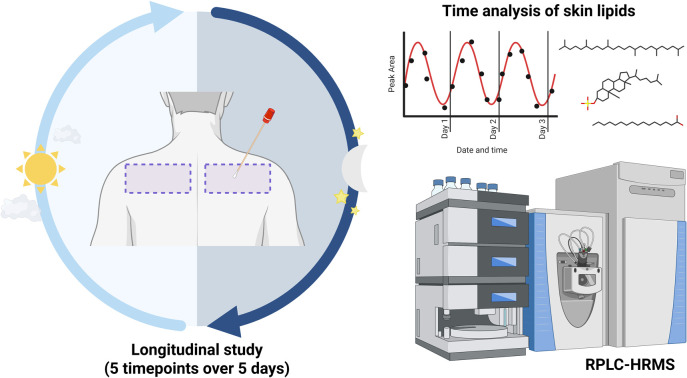

## Introduction

The circadian rhythm is a 24 h oscillatory cycle that the human body follows to synchronise internal metabolic functions with external changes.^1–5^ Circadian rhythms are governed by the ‘master clock’, the suprachiasmatic nucleus (SCN) of the anterior hypothalamus in the brain, which signals responses depending on light changes detected at the retinae,^1,2,4,5^ but are also controlled by tissue-specific peripheral clocks receptive to a wider variety of triggers, such as coexisting hormonal changes,^2,6^ diet, and activity level.^2,4,7–13^ Routines executed by both clocks are communicated using electrical and endocrinal signals.^12,14^

Transcriptional research of *Neurospora*, *Drosophila*, *mouse*, and *homo sapiens* indicated that up to 10% of gene expression in any tissue is rhythmical.^15^ The circadian rhythms influence molecules in living organisms^16–25^ and are controlled by cellular activities, such as protein transcription and translation, cellular metabolism, and cellular oxidation and reduction.^22,26–28^ Like properties of other organs of the body, skin and its associated properties, such as transepidermal water loss, keratinocyte proliferation, blood flow, permeability, pH, and temperature, have been demonstrated to have rhythmic variations.^5,9,29–35^

At the molecular level, the skin is a complex organ with a multi-faceted lipid composition, comprising of molecules that are directly correlated to internal metabolic processes, microbial activity, and external environmental exposures.^36^ The skin surface lipids (SSL) originate from two main cutaneous sources: the sebaceous glands and stratum corneum (SC).^37^ The sebaceous glands excrete sebum onto the skin surface with a relative composition of 30–50% triacylglycerols or diacylglycerols, 15–30% free fatty acids, 12–20% squalene, 26–30% wax esters, 3–6% cholesterol esters, and 1.5–2.5% cholesterol,^38,39^ and the epidermal lipids of the stratum corneum have a relative composition of 45–50% ceramides, 10–15% free fatty acids, 25–27% cholesterol, 10% cholesteryl esters, and 2–5% cholesterol sulfate.^40–42^

The novelty and ease of SSL analysis have garnered interest in *omics* communities, where the number of applications has rapidly grown in the last 5 years.^38^ Recently, it has been shown that skin metabolomics can potentially be used to direct the clinical diagnostics of Parkinson's disease,^39,43,44^ Alzheimer's disease,^44^ malaria,^45,46^ leprosy,^47^ and COVID-19.^48,49^ Many factors, including biological and lifestyle factors, *e.g.*, seasonal variation, age, ethnicity, and diet, affect SSL production and composition.^38,50–57^ To the best of our knowledge, no studies to date have investigated the effects of circadian rhythm on SSL composition.

We investigated the skin lipidome from eight healthy participants longitudinally using an untargeted reversed-phase liquid chromatography-mass spectrometry (RPLC-MS) lipidomics approach. The participants gently rubbed two cotton swabs on their upper backs across five timepoints (7:00–8:00, 11:00–12:00, 15:00–16:00, 19:00–20:00, 23:00–00:00; *i.e.*, 4/8 h windows) per day for five consecutive days using our home sampling protocol. We report the findings from this analysis (on a group-mean and individual-mean bases) focused on two main objectives:

(1) Detect and putatively identify metabolites with a circadian trend.

(2) Evaluate the ways in which temporal metabolic changes (and other potential confounders) may impact the development of clinical diagnostics.

## Results and discussion

### Data-driven classification of circadian skin metabolites

A total of 208 samples were obtained from 8 healthy participants (SI Table S1). The skin cleaning protocol using ultrapure water and gauze removed ∼80% of SSL (SI Table S2). With the exception of the 7:30 timepoint with 8 hours, all other timepoints had the same number of hours for SSL accumulation across participants (SI Table S7).

The home sampling approach employed in this study offers a convenient sample collection option. Clinical testing conducted with patients sampling from their home, which was prominent in the diagnostics of COVID-19, is a method that allows patients to sample both non-invasively and readily without transportation to a clinical location. This study shows that skin-surface lipidomics on self-collected samples from eight different individuals were plausible and yielded many SSLs for analysis.

A total of 4337 features were robustly measured using our LC-MS methodology. Representative LC-MS chromatograms are shown in SI Fig. S4. Relevant feature annotations across the analyses have been tabulated in SI Table S6. Using the GNPS and SIRIUS thresholds described for structure identification, 5.21% (226 features) have Level 2 annotations (SI Table S10).‡‡Note that some of the identifications include contaminants and overlapping identifications across features. Some of the identifications are considered improbable as they are likely to be environmental (exogenous) origins. Of the few annotations overall, the representation of squalene and triacylglycerols are characteristic of sebum;^58,59^ whereas, sphingolipids and ceramides are characteristic of stratum corneum,^40–42^ with both sources sharing the fatty acids.

### Metabolites following circadian (24 h) rhythmicity

**Fig. 1:**
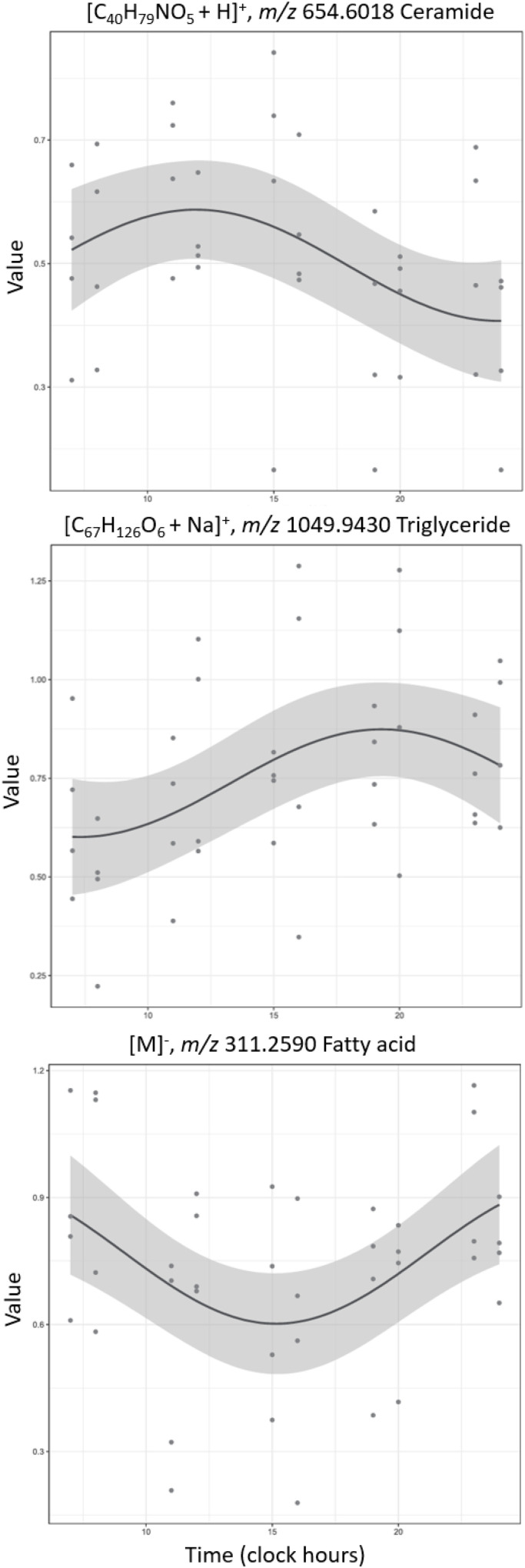
Example cosinor curves of metabolites with putative annotations that match expected SSL classes (significant by *p*-value). The curved line and the shaded area represent the fitted periodic sinusoidal curve and the 95% confidence band.

Using DiscoRhythm,^60^ we identified few metabolites showing 24 h rhythmicity by cosinor analysis (*p*-value ≤ 0.05, Table 1). Example cosinor curves of different SSL across all participants are shown in Fig. 1. Interestingly, none of the metabolites showed rhythmicity by Lomb–Scargle analysis. Further, when correcting for multiple comparisons testing using FDR *q*-values, only six metabolites were considered significant for participant 4 (Table 1).

**Table 1:** Metabolites showing 24 h rhythmicity according to cosinor analysis *p*-values, shown as mean values across the participant group and individual results

Participant	% of total metabolites showing 24 h rhythmicity by cosinor analysis by *p*-value (# of significant metabolites)^a^		
	RPLC+	RPLC−	Combined
Overall	0.47 (12)	0.95 (17)	0.67 (29)
1	5.76 (145)	5.48 (97)	5.65 (242)

2	26.43 (662)	5.57 (99)	17.77 (761)
3	2.93 (73)	5.95 (105)	4.18 (178)
4^a^	23.62 (592)	11.32 (200)	18.53 (792)
5	2.00 (50)	5.65 (100)	3.51 (150)
6	1.04 (26)	8.46 (148)	4.10 (174)
7	2.59 (65)	10.45 (184)	5.83 (249)
8	5.84 (146)	2.21 (39)	4.34 (185)

**Averaged across participants**	8.78 (220)	6.89 (122)	7.99 (341)

a When using FDR *q*-values, only six metabolites for participant 4 in RPLC+ are considered rhythmic (*q*-value ≤ 0.05).

The cosinor analysis highlighted the wide heterogeneity within subjects, which consolidates the need for the longitudinal nature of the study to allow isolated within-subject data analysis and interpretation. Overall, combining the data sets in a mixed-effects cosinor analysis demonstrated the skin lipidome to have a combined 24 h rhythmicity of 0.67%; whereas, individuals showed 3.51–18.53% of total detected features that demonstrated rhythmicity, with a combined average of 7.99% (Table 1). The feature annotations that trended across all participants are presented in SI Table S6.

### Targeted analysis of sebum and skin specific metabolites

**Fig. 2:**
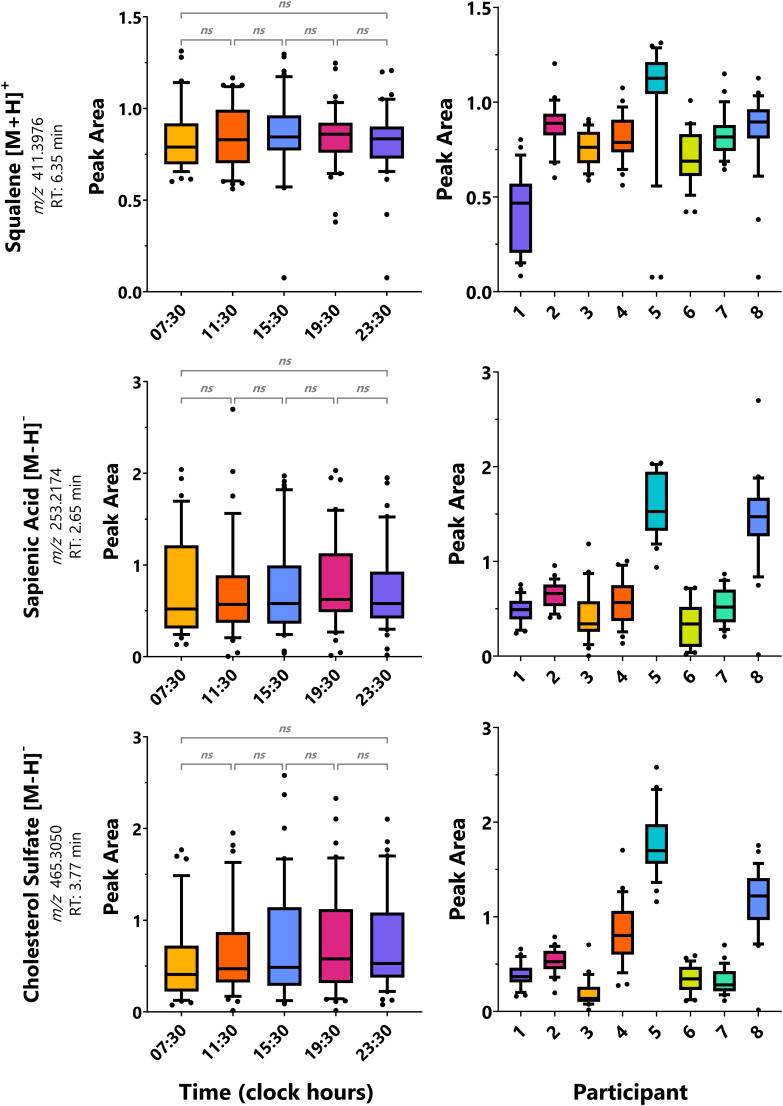
Time- and participant-dependent trends of sebaceous and stratum corneum-specific metabolites. Range bars represent values within the 10–90 percentiles. Across participants, there were no significant (*p*-value ≤ 0.05) pairs for timepoints; whereas, across timepoints, multiple significant pairs for participants were identified by one-way ANOVA with Tukey's multiple comparisons correction (SI Table S9).

Three analytes were investigated due to their established specificity to sebaceous gland or stratum corneum production: squalene (sebum), sapienic acid (sebum), and cholesterol sulfate (stratum corneum). Using one-way ANOVA on the unadjusted peak areas of each analyte on a group level (Fig. 2) and on an individual level, none of the molecules or timepoints were found significant except for squalene for participant #3 (SI Table S8).

For cosinor and Lomb–Scargle analysis, none of the analytes were rhythmic at a group level. On an individual level, none of the analytes showed 24 h rhythmicity except for squalene by cosinor analysis that was found to be significant by *p*-value for participants #2 and 4 with an acrophase at 18:06 and 20:03, respectively (SI Table S8). This acrophase is earlier than those reported for plasma squalene levels of 24:00–04:00.^24^

The effect of the participant was also investigated by combining the timepoint data and classifying by participant. Here, ANOVA demonstrated the significance of participant differences (Fig. 2; SI Table S9). Large interindividual variations of squalene, sapienic acid, and cholesterol sulfate concentrations are seen, highlighting either differences in sampling and/or individual skin lipidomes.

### Impacts on clinical research

**Fig. 3:**
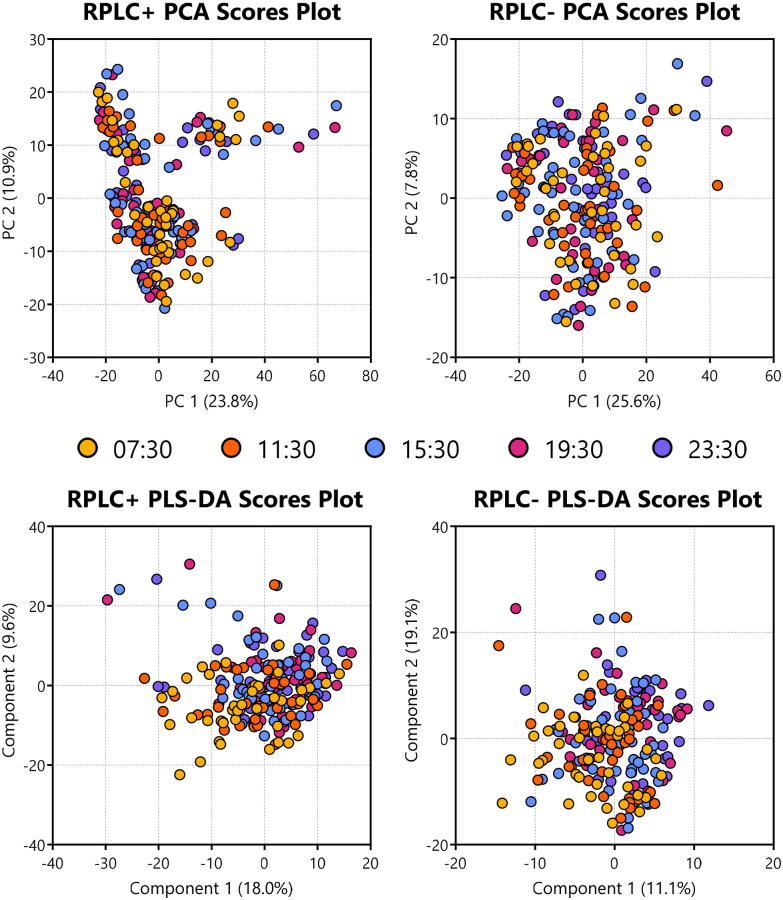
Score plots for the PCA and PLS-DA analyses using a five-way input of timepoints, showing little/no separation.

As a combined cohort data set, 100% of features were found to be insignificant based on timepoint by ANOVA. PCA and PERMANOVA analyses showed no separation by timepoint (Fig. 3 and SI Table S5). PLS-DA analysis also showed no clear clustering by timepoints (Fig. 3) with a cross validation that indicated overfitting (SI Fig. S3). Therefore, it was concluded that there is a negligible contribution of timepoint differences at a group level.

The same processing was performed on an individual basis. Here, half of the participants showed significant differences in their SSL profiles at the 7:30 timepoint against the other timepoints (11:30, 15:30, 19:30, 23:30) by PCA PERMANOVA *p*-values (one participant by FDR *q*-values). Significant features detected by ANOVA (FDR) ranged from 0–4.74% of total features. This difference in analysis results shows at an individual level, participants show variability immediately after the nadir phase of their circadian cycles.

### Personalised routines

The strength of this study is home sampling,^61^ which is more convenient for the clinical sampling of skin swabs. Compared to conventional sleep studies, a key difference of this study is no participant conditioning prior to sampling as well as no harmonisation of light cycles, diets, sleep or environment was induced, which could have potentially synchronised their body clocks and enabled easier interpretations of SSL molecular behaviour. However, within a clinical context, this pre-conditioning step would not be done and therefore, this study design mimics typical clinical recruitment, where any person with their individualised routines would be sampled as presented.

These data show that as a combined cohort, the interindividual variation is larger than the circadian variation, and therefore, the time of sampling does not need to be taken into account (as a potential confounder) during SSL analysis. Interestingly, on an individual level, a significant portion of the SSL showed rhythmicity (average 7.99% of all features detected). The difficulty of metabolite identifications in untargeted analyses limits our ability to confidently report which molecules are showing circadian rhythmicity.

During the identification workflow, confident hits flagged multiple exogenous contaminants that were introduced through personal care routines (*i.e.*, shampoo, conditioners, shower gels), highlighting the complexities of skin surface analysis. These features were putatively annotated as ionic surface-active agents (surfactants) (SI Fig. S5). Due to their amphiphilic nature and native formal charges, they were detected at high intensities in this data set.

As further evidence, we confidently identified the presence of behentrimonium chloride, cocamidopropyl betaine, and stearamidopropyl dimethylamine. These analytes were not effectively blank subtracted in our processing workflow since they only appear on participants that used products containing these surfactants. These contaminants, which are difficult to control in terms of the multitude of products that exist and when/how much an individual uses, could bias normalization approaches and impose matrix effects. It is proposed that the surfactant contaminant features identified in this study should be subtracted analytically (*e.g.*, through exclusion lists to avoid their MS analysis) or controlled during sample collection by avoiding the application of surfactant-based products to both the sampling area and the hair for future studies.

## Experimental

### Materials

The chemicals and materials used in this study were as follows: sterile cotton swabs in plastic applicators (Deltalab, Spain), Hypacover gauze swabs 8 ply (Safety First Aid Group, UK), microcentrifuge tubes (1.5/2 mL) (Eppendorf, UK), glass dram vials (7/28 mL) with polypropylene caps (SAMCO, UK), fixed insert (300 µL) amber LC vials (Thermo Scientific, USA), PTFE screw caps (Agilent, USA), LC-MS grade (≥99%) ammonium formate (VWR Chemicals, USA) and analytical reagent grade (≥99%) ammonium acetate (Fisher Scientific, USA), LC-MS grade solvents 2-propanol (Fisher Scientific, USA), acetonitrile (Supelco, USA), methanol (Supelco, USA), and formic acid (Fisher Scientific, USA), ultrapure water (Veolia, UK), and Pierce™ LTQ Velos ESI Positive/Negative Ion Calibration Solutions (Thermo Scientific, USA). The instruments and equipment used were as follows: 20–200 µL and 100–1000 µL micropipettes (Starlab, UK), vortex mixer (IKA, Germany), centrifuge (Sciquip, UK), sonic baths (Sonicor, USA; Thermo Scientific, USA), vacuum centrifuge (Eppendorf, UK), and Ultimate 3000 UHPLC (Thermo Scientific, USA) coupled to a Q-Exactive™ Plus Hybrid Quadrupole-Orbitrap™ mass spectrometer (Thermo Scientific, USA).

### Participants and sample collection

In this longitudinal experiment, skin surface samples were collected from eight consenting participants (University of Manchester ethical approval: 2024-19932-38117) over five 24 h periods (six calendar days) between November 2024–January 2025. The method of sampling was instructed home self-sampling, in which participants were given organised packs of sampling materials and instructed to sample over five timepoints during the day, each covering 4 h windows, excluding an 8 h window for sleeping. Timepoints covered: early morning [7:00–8:00], midday [11:00–12:00], afternoon [15:00–16:00], evening [19:00–20:00], night/before sleep [23:00–24:00]. Between the indicated timepoints, participants sampled their upper back twice using a sterile cotton swab, rubbing and rotating the swab for 30 seconds; one swab on their left and one swab on their right upper back (SI Fig. S1). All left samples were used for positive mode, and all right samples were used for negative mode. The swabs were then returned to their transport tube and kept at ambient temperature until their collection by the research team within 36 hours of sampling, a time window within which SSLs have been found to be stable at room temperature.^61^ Following sampling, participants used gauze with ultrapure water to first clean the sampling area and then dry gauze to pat it dry, thereby “resetting” the sampling area for the next timepoint. The data supporting the skin cleaning protocol were collected from three consenting participants (University of Manchester ethical approval: 2022-9029-24560; SI Table S2). Samples and questionnaires (described below) were submitted daily. After collection by the research team, the swab tips were immediately snapped into 2 mL microcentrifuge tubes and stored at −80 °C until extraction.

All participants recorded their exact sampling and washing times, sleep times, a food diary, and a list of any personal care products applied to the hair and body during the day in daily questionnaires (SI Table S3). During the sampling period, participants avoided strenuous exercise, limited alcohol consumption to a maximum of one unit per day, and avoided sampling during the period of menstruation, which are known to affect skin lipid production.^18,62,63^

### Sample extraction

Samples were equilibrated at ambient temperature (approx. 21 °C). Methanol (1 mL) was added to each sample, and then the lipids were extracted from the swab by vortexing (30 s) and sonicating (30 min) at ambient temperature. No stabilising buffers were used. The cotton swab was removed from the tube using tweezers, followed by centrifugation (15 min) at 12 000*g*. 600 µL of each sample extract was transferred to a new microcentrifuge tube (1.5 mL), and a further 100 µL of each sample extract was combined in a glass dram vial, followed by vortexing (30 s) to create a pooled quality control (QC) sample. The entire volume of pooled QC was split into 600 µL fractions. All individual sample extracts and QC aliquots were subsequently vacuum concentrated at ambient temperature to dryness (∼3 h). The dried pellets were stored at −80 °C until analysis.

### Sample reconstitution and analyses

The dried samples were removed from the −80 °C freezer and equilibrated to room temperature. The samples were reconstituted in 100 µL methanol, followed by vortexing (30 s), sonication (30 min) and centrifugation (15 min) at 12 000*g*. 80 µL of supernatant was submitted for LC-MS analysis.

Pooled QC samples were used to check analytical reproducibility and were injected at the beginning of each analytical batch (*n* = 5), every 5th injection, and at the end of the sequence (*n* = 5). Blank swabs were analysed in duplicate at the beginning and end of the sequence for blank subtraction purposes. The 208 samples were analysed in a randomised order and run as a single analytical sequence with single injections. All samples were injected within 24 h of reconstitution to maintain integrity.

### LC-MS parameters

An Ultimate 3000 UHPLC (Thermo Scientific, USA) coupled to a Q-Exactive™ Plus Hybrid Quadrupole-Orbitrap™ mass spectrometer (Thermo Scientific, USA) was used to collect data in positive and negative ionisation modes separately. Chromatographic separation was performed using an ACQUITY UPLC CSH C18 column (1.7 µm, 2.1 mm × 100 mm) with an Acquity UPLC CSH C18 VanGuard pre-column heated to 55 °C. For positive ionisation mode, mobile phase A was acetonitrile: water (*v*/*v* 60 : 40) with 0.1% formic acid made to a 10 mM solution of ammonium formate, and mobile phase B was isopropanol: acetonitrile (*v*/*v* 90 : 10) with 0.1% formic acid made to a 10 mM solution of ammonium formate. For negative ionisation mode, mobile phase A was acetonitrile: water (*v*/*v* 60 : 40) with 10 mM ammonium acetate, and mobile phase B was isopropanol: acetonitrile (*v*/*v* 90 : 10) with 10 mM ammonium acetate. The injection volume utilised was 5 µL. The flow rate was set at 0.55 mL min^−1^, and the gradient elution began at 10% B with a hold for 1 min before increasing to 70% B at 3.8 min and 95% B at 8 min. At 9.1 min, the gradient was lowered to 10% B and maintained for 4 min to equilibrate the column. The needle was washed with 100% IPA between samples.

MS calibration was performed by infusing Pierce™ LTQ Velos ESI Positive/Negative Ion Calibration Solutions (Thermo Scientific, USA) prior to analysis. The Q-Exactive™ Plus Hybrid Quadrupole-Orbitrap™ MS was operated in positive and negative HESI modes in a data-dependent MS/MS spectra acquisition method. The ion source conditions were as follows: spray voltage, 3.5 kV (positive), 2.5 kV (negative); sheath gas flow rate, 50 arbitrary units; aux gas flow rate, 13 arbitrary units; sweep gas flow rate, 3 arbitrary units; capillary temp, 320 °C; S-lens RF level, 50 (positive), 70 (negative); Aux gas heater temperature, 425 °C. The following acquisition parameters were used for MS1 analysis: resolution, 70 000, AGC target, 3e6; Maximum IT, 100 ms; scan range 150–2250 *m*/*z* (positive), 100–1500 *m*/*z* (negative); spectrum data type, profile. Data-dependent MS/MS parameters: resolution, 35 000; AGC target, 1 × 10^5^; maximum IT, 50 ms; loop count, 5; TopN, 5; isolation window, 4.0 *m*/*z*; fixed first mass, -; (N)CE/stepped nce, 30; spectrum data type, profile; minimum AGC target, 8 × 10^3^; intensity threshold, 1.6 × 10^5^; exclude isotopes, on; dynamic exclusion, 10.0 s.

### Data pre-processing and deconvolution

LC-MS .raw files were converted to .mzML format using MSconvert, Proteowizard.^64^ MS data processing was performed using MZmine 4.5.0 ^65^ for peak extraction, alignment, and blank subtraction (SI Table S4).

The resultant matrices were .csv files containing deconvolved features as rows and samples as columns (6874 features for positive; 4669 features for negative). Features present in ≤75% of total QC injections were manually removed (2150 features removed for positive; 1757 for negative). The peak areas were then normalised with reference to the pooled QC using LOESS correction.^66^ The resulting peak tables had 4724 features for positive and 2912 features for negative.

Missing values in the original data were replaced by one-fifth of the smallest positive value, which is considered the detection limit. Features with peak intensities exceeding 20% relative standard deviation (RSD) in QC samples were further removed (2171 features removed for positive; 1128 for negative). The remaining features (2553 for positive; 1784 for negative) were used for data analysis.

### Statistical analysis

#### Analytical checks

Principal component analysis (PCA) of the processed data was performed to assess analytical performance (SI Fig. S2). PCA showed a tight QC clustering around the origin (0,0). All samples are dispersed around the QCs with little/no discrimination in unsupervised analysis. QC data were excluded for the rest of the data analysis.

#### Circadian behaviour analysis

For the analysis of metabolite rhythmicity (*n* = 8 biological samples with *n* = 5 technical replicates for each timepoint), the unadjusted analyte peak areas were analysed using the DiscoRhythm web application 1.2.1.^60^ This analysis was performed on group-mean and individual-mean data. Due to the uneven spacing of the timepoints, JTK_cycle and ARSER analyses were not possible; therefore, cosinor and Lomb–Scargle analyses were used. The analytes indicating 24 h rhythmicity through a *p*-value ≤ 0.05 were putatively identified (SI Table S6).

#### Impact to clinical diagnostics assessment

To direct the analytical processing used in this assessment, a review of the data scaling approaches used in modern skin diagnostics literature was performed. Data scaling techniques applied in the majority of research were identified to be: normalisation of peak area by total ion count (sum), log_10_ transformation and Pareto scaling, and therefore, this scaling was similarly applied to these data.

Statistical analysis was performed using MetaboAnalyst 6.0.^67^

Features were analysed using PCA with PERMANOVA (Fig. 3 and SI Table S5) and partial least squares discriminant analysis (PLS-DA) using a five-class input of the timepoints with 5-fold cross-validation (Fig. 3 and SI Fig. S3). One-way ANOVA was performed across all groups to identify significant features with an FDR *q*-value ≤ 0.05.

#### Analysis of squalene, sapienic acid, and cholesterol sulfate

The unadjusted analyte peak areas were first analysed using one-way ANOVA to check for any significant differences between timepoints.

If any feature was flagged as significant by *p*-value, it was followed up with Tukey's multiple comparison testing. This was performed at a group and individual level.

#### Feature annotation

Metabolomics Standards Initiative (MSI) guidelines^68^ and International Lipid Classification and Nomenclature Committee (ILCNC)^69^ guidelines were adhered to for metabolite annotations (SI Table S6).

For all compounds, the identification of features was conducted with two separate MS/MS-based identification software: SIRIUS 6.1.1 ^70^ and GNPS,^71^ where annotations are Level 2/3. Feature identifications in GNPS with a cosine score of ≥0.7 and ≥6 shared peaks were accepted. Feature identifications in SIRIUS with a confidence score ≥0.7 were accepted. Molecular formula determinations in SIRIUS with a ZODIAC^72^ score ≥99%, ≥80% of peak intensity explained by SIRIUS, and a ≥20 tree score were accepted. Compound classes with a confidence of ≥80% were also accepted in SIRIUS in the absence of a suitable formula.

For their relative quantification, squalene and sapienic acid have been Level 1 annotated using chemical standards, and cholesterol sulfate has been Level 2 annotated. In terms of detected contaminants, behentrimonium chloride, cocamidopropyl betaine, and stearamidopropyl dimethylamine have been Level 1 annotated using cosmetic raw materials.

## Conclusions

We present a longitudinal data set capable of identifying circadian skin lipid behaviour using non-invasive home sampling using cotton swabs. Due to the longitudinal nature of this study, it was possible to investigate for circadian rhythmicity and timepoint differences at both a group and individual level. By combining the healthy cohort, few/no temporal changes were found (≤0.67% of features), but this is thought to be a poor representation as individually, 3.51–18.53% of features were considered rhythmic which suggests that this number should be higher. With participant conditioning prior to enrollment and more control of their environment/diet, it is hypothesized that the number of features across a cohort would increase.

On the other hand, in terms of how current research groups are conducting their skin disease diagnostic modelling^39,43,48,49^ and how clinical recruitment is typically performed (*i.e.*, a lack of prior patient conditioning), our data show the contribution of the circadian rhythm on metabolites would be negligible. As most studies compare groups of participants instead of individuals, person to person difference in circadian rhythm observed in our work, does not affect those data.

## Author contributions

Caroline Géhin: conceptualisation, methodology, formal analysis, investigation, visualization, writing – original draft, writing – review & editing. Amanda Witter: methodology, formal analysis, investigation, visualization, writing – original draft, writing – review & editing. Lu Wang: conceptualisation, methodology. Perdita Barran: writing – review & editing. Stephen Fowler: writing – review & editing. Drupad Trivedi: conceptualisation, methodology, writing – review & editing, supervision.

## Conflicts of interest

There are no conflicts of interest to declare by any of the authors.

## Supplementary Material

AN-150-D5AN00665A-s001

AN-150-D5AN00665A-s002

AN-150-D5AN00665A-s003

## Data Availability

All raw data are available in open source (.mzml) format on public repository –MetaboLights with study accession number: MTBLS12607. All our methods used to deconvolve and analyse data have been reported in the manuscript as well as in supplementary information (SI). Supplementary information is available. See DOI: https://doi.org/10.1039/d5an00665a.
