# Supplementary material for: Investigating the effects of circadian rhythm on the human skin lipidome
Source: Analyst. 2025 Oct 14;150(22):4962–71. doi: 10.1039/d5an00665a (PMC12541903; doi:10.1039/d5an00665a)

Supplementary Information (SI) for Analyst.
This journal is © The Royal Society of Chemistry 2025

Electronic Supplementary Information (ESI)

Tables:

ESI Table 1: Main demographic characteristics of study participants.

**Demographics**	Mean ± SD or No. (%)
**Participants**	8
**Age (years)**	28.63 ± 5.01
**Sex (at birth)**	Female (5; 62.5%), Male (3; 37.5%)
**BMI (kg/m2)**	21.17 ± 3.73
**Ethnicity**	Mixed (1; 12.5%)<br>White (3; 37.5%)<br>Indian (3; 37.5%)<br>Hispanic/Latin American (1; 12.5%)
**Smoking status**	Never smoke (7; 87.5%)<br>Ex-smoker (1; 12.5%)
**Drinking status**	Never (2; 25.0%)<br>Once a week (3; 37.5%)<br>1-3 times a month (3; 37.5%)

**Full participant details given in ESI Table 1a (attached as .csv)*

ESI Table 2: Skin cleaning protocol data.

	**Sebumeter® measurements (AU)**											
	**CRS01**				**CRS02**				**CRS03**			
	Rep 1	Rep 2	Rep 3	**Avg**	Rep 1	Rep 2	Rep 3	**Avg**	Rep 1	Rep 2	Rep 3	**Avg**
	Pre-wash											
Left back	5	6	6	5.7	6	3	4	4.3	20	12	6	12.7
Right back	2	6	2	3.3	6	9	2	5.7	18	13	19	16.7
	Post-wash											
Left back	1	2	3	2.0	2	1	3	2.0	4	0	1	1.7
Right back	0	1	1	0.7	2	1	1	1.3	8	5	1	4.7
	**%Reduction**			70.4	**%Reduction**			66.7	**%Reduction**			78.4

	**Sebumeter® measurements (AU)**											
	**CRS01**				**CRS02**				**CRS03**			
	Rep 1	Rep 2	Rep 3	**Avg**	Rep 1	Rep 2	Rep 3	**Avg**	Rep 1	Rep 2	Rep 3	**Avg**
	Pre-wash											
Left back	5	6	3	4.7	17	20	16	17.7	5	7	10	7.3

Right back	3	4	6	4.3	15	16	17	16.0	6	3	8	5.7
	Post-wash											
Left back	0	1	1	0.7	2	2	3	2.3	0	3	0	1.0
Right back	1	0	1	0.7	2	3	2	2.3	2	2	0	1.3
	**%Reduction**			85.2	**%Reduction**			86.1	**%Reduction**			82.1

Calculated average %reduction in Sebumeter® measurements after skin cleaning = 78.1%

ESI Table 3: Daily questionnaire data summary (*Attached as separate .csv file).*

ESI Table 4: MZmine parameters used.

	**RPLC+**	**RPLC-**
1. Crop filtering	RT range: 0.40 - 10.00 min	
2. Mass detection using MS absolute intensities	MS1: 1.0E5<br>MS2: 1.0E4	
3. ADAP chromatogram builder	Min con. scans: 4<br>Min int. for con. scans: 2.0E4<br>Min. abs. height: 1.0E5<br>*m/z* tol.: 0.0020 *m/z* or 5.00 ppm	
4. Savitzky Golay smoothing applied.		
5. Chromatogram deconvolution using local min. feature resolver	Chrom. threshold: 75.0%<br>Min. RT range: 0.080<br>Min. rel. height: 0.0%<br>Min. abs. height: 1.0E5<br>Min. ratio of peak top/edge: 1.80<br>Peak dur. range: 0.00-2.40 min<br>Min. scans: 4	Chrom. threshold: 84.4%<br>Min. RT range: 0.050<br>Min. rel. height: 0.0%<br>Min. abs. height: 1.0E5<br>Min. ratio of peak top/edge: 1.80<br>Peak dur. range: 0.00-1.51 min<br>Min. scans: 4
6. 13C isotope filter	*m/z* tol.: 0.0015 *m/z* -3.00 ppm<br>RT tol.: 0.04 min<br>Max. charge: 2<br>Most intense isotope as the representative isotope	
7. Join aligner	*m/z* tol.: 0.0015 *m/z* or 5.00 ppm<br>*m/z* weight: 3	m/z tol.: 0.0015 m/z or 5.00 ppm<br>m/z weight: 3

	RT tol.: 0.25 min<br><br>RT weight: 1<br><br>No requirement of charge state or ID and no comparison of isotope pattern	RT tol.: 0.10 min<br><br>RT weight: 1<br><br>No requirement of charge state or ID and no comparison of isotope pattern
8. Peak list row filter with a minimum of 5 samples or 2% of samples per aligned feature. Only keep features with 13C.		
9. Gap filling using peak finder algorithm	Int. tol.: 20.0%<br><br>*m/z* tol.: 0.0015 *m/z* or 5.00 ppm<br><br>RT tol.: 0.25 min<br><br>Min. scans: 2	Int. tol.: 20.0%<br><br>*m/z* tol.: 0.0015 *m/z* or 5.00 ppm<br><br>RT tol.: 0.10 min<br><br>Min. scans: 2
10. Duplicate peak filtering	*m/z* tol.: 0.0008 *m/z* or 1.50 ppm<br><br>RT tol.: 0.06 min	*m/z* tol.: 0.0008 *m/z* or 1.50 ppm<br><br>RT tol.: 0.04 min
11. Feature list blank subtraction by average area of all blanks in the sequence for all features with a fold change increase of 100.0%.		

ESI Table 5: PERMANOVA comparisons of RPLC+ and RPLC- PCA, showing that no groups have significant ($p \leq 0.05$ and q (FDR) ≤ 0.05) relationships.

RPLC+	F.Model	R^2	P-value	FDR Q-value
11:30 vs 15:30	0.05940	0.00069	0.94700	0.99400
11:30 vs 19:30	0.11237	0.00291	0.78300	0.99400
11:30 vs 23:30	0.18759	0.00240	0.81400	0.99400
11:30 vs 7:30	0.05885	0.00075	0.93500	0.99400
15:30 vs 19:30	0.20213	0.00234	0.81300	0.99400
15:30 vs 23:30	0.17086	0.00198	0.82600	0.99400
15:30 vs 7:30	0.07158	0.00083	0.93100	0.99400
19:30 vs 23:30	0.00365	0.00005	0.99400	0.99400
19:30 vs 7:30	0.43491	0.00554	0.64700	0.99400
23:30 vs 7:30	0.39243	0.00501	0.63300	0.99400

RPLC-	F.Model	R^2	P-value	FDR Q-value
11:30 vs 15:30	0.19254	0.00226	0.78700	0.96900
11:30 vs 19:30	0.03677	0.00097	0.91100	0.96900
11:30 vs 23:30	0.24046	0.00311	0.72300	0.96900

11:30 vs 7:30	0.03078	0.00040	0.96900	0.96900
15:30 vs 19:30	0.02884	0.00034	0.96500	0.96900
15:30 vs 23:30	0.33316	0.00386	0.70800	0.96900
15:30 vs 7:30	0.38946	0.00456	0.61800	0.96900
19:30 vs 23:30	0.20580	0.00263	0.77100	0.96900
19:30 vs 7:30	0.19356	0.00251	0.79400	0.96900
23:30 vs 7:30	0.25691	0.00333	0.75700	0.96900

ESI Table 6: Putative annotations of significant features showing 24-hr rhythmicity. Text in red indicates a significant hit.

#	Precursor *m/z* [ion]	RT (min)	Elemental composition (SIRIUS)	Median mass error (ppm)	Peak int. explained (%)	Tree score	SIRIUS structure ID (confidence)	SIRIUS compound class (confidence %)	Acrophase (hr)	Cosinor analysis		Lomb-Scargle analysis	
										P-value	FDR Q-value	P-value	FDR Q-value
1	277.0833 $[M+K]^+$	0.68	$C_{13}H_{18}0_4$	3.210	94.49	303.81	N/A	Carboxylic acids and derivatives (81%)	4.38	0.041	1.0	0.93	1.0
2	874.5143 $[M+NH_4]^+$	3.02	$C_{44}H_{72}0_{16}$	1.341	97.44	275.94	N/A	Steroids and steroid derivatives (89%)	10.93	0.037	1.0	0.84	1.0
3	1228.8690 $[M+H]^+$	3.90	$C_{64}H_{113}N_{11}O_{12}$	1.490	90.14	38.69	N/A	Amino acids, peptides, and analogues (97%)	13.00	0.030	1.0	0.83	1.0
4	1056.2170 $[M]^+$	4.04	N/A	N/A	N/A	N/A	N/A	O-glycosyl compounds (82%)	13.09	0.035	1.0	0.86	1.0
5	1033.7026 $[M]^+$	4.04	N/A	N/A	N/A	N/A	N/A	Amino acids, peptides, and analogues (99%)	12.61	0.011	1.0	0.51	1.0
6	1011.6890 $[M]^+$	4.06	N/A	N/A	N/A	N/A	N/A	Peptidomimetics (83%)	12.64	0.020	1.0	0.70	1.0
7	769.5451 $[M-H_20+H]^+$	4.17	$C_{49}H_{74}N_2O_6$	3.441	82.65	85.02	N/A	Carboxylic acids and derivatives (97%)	9.96	0.018	1.0	0.62	1.0
8	728.5887 $[M-H_20+H]^+$	4.47	$C_{42}H_{84}NO_7P$	6.549	95.64	64.33	PE(P-37:0) (0.763)	1-(1Z-alkenyl),2-acylglycerophosphoethanolamines (92%)	13.38	0.046	1.0	0.93	1.0
9	654.6018 $[M+H]^+$	5.77	$C_{40}H_{79}NO_5$	3.565	92.71	170.74	N/A	Ceramides (100%)	11.92	0.043	1.0	0.89	1.0
10	600.5338 $[M+Na]^+$	6.23	$C_{37}H_{71}NO_3$	2.052	90.85	108.51	N/A	Fatty acyls (81%)	9.31	0.024	1.0	0.70	1.0
11	711.7032 $[M+H]^+$	7.14	$C_{43}H_{90}BNO_5$	2.592	90.27	106.44	N/A	N-acyl amines (83%)	2.24	0.037	1.0	0.90	1.0

12	1049.9430 $[M+Na]^+$	9.25	$C_{67}H_{126}O_6$	3.258	87.79	73.06	N/A	Triacylglycerols (100%)	19.29	0.037	1.0	0.85	1.0
13	215.0325 $[M]^-$	0.42	N/A	N/A	N/A	N/A	N/A	Organoheterocyclic compounds (100%)	6.23	0.031	1.0	0.83	1.0
14	320.9975 $[M]^-$	0.46	N/A	N/A	N/A	N/A	N/A	Carboxylic acids and derivatives (99%)	5.72	0.0032	1.0	0.27	1.0
15	156.9900 $[M]^-$	0.47	N/A	N/A	N/A	N/A	N/A	Organooxygen compounds (90%)	4.60	0.00059	1.0	0.11	1.0
16	337.2391 $[M]^-$	0.67	N/A	N/A	N/A	N/A	N/A	Fatty acyls (98%)	3.87	0.040	1.0	0.93	1.0
17	312.2267 $[M]^-$	0.68	N/A	N/A	N/A	N/A	N/A	Fatty acyls (94%)	4.2	0.025	1.0	0.82	1.0
18	311.2597 $[M]^-$	1.49	N/A	N/A	N/A	N/A	N/A	Fatty acids and conjugates (98%)	3.15	0.025	1.0	0.83	1.0
19	399.2758 $[M]^-$	1.64	N/A	N/A	N/A	N/A	N/A	Fatty acid esters (97%)	5.03	0.037	1.0	0.90	1.0
20	438.2326 $[M-H]^-$	2.26	$C_{23}H_{37}NO_5S$	-1.160	86.57	48.82	N/A	Organic compounds (100%)	20.17	0.023	1.0	0.71	1.0
21	289.2178 $[M]^-$	2.48	N/A	N/A	N/A	N/A	N/A	N/A	3.00	0.0084	1.0	0.53	1.0
22	341.2703 $[M]^-$	2.78	N/A	N/A	N/A	N/A	N/A	Fatty acyls (92%)	2.77	0.042	1.0	0.93	1.0
23	822.7577 $[M]^-$	6.79	N/A	N/A	N/A	N/A	N/A	Ceramides (91%)	23.23	0.034	1.0	0.82	1.0
24	738.6997 $[M]^-$	6.79	N/A	N/A	N/A	N/A	N/A	Ceramides (100%)	22.99	0.043	1.0	0.88	1.0
25	782.7263 $[M]^-$	6.81	N/A	N/A	N/A	N/A	N/A	Ceramides (100%)	0.22	0.017	1.0	0.64	1.0
26	1087.9702 $[M]^-$	7.17	N/A	N/A	N/A	N/A	N/A	Fatty amides (80%)	1.24	0.036	1.0	0.87	1.0
27	1086.967 $[M]^-$	7.17	N/A	N/A	N/A	N/A	N/A	Sphingolipids (93%)	1.34	0.037	1.0	0.89	1.0

2<br>8	1114.998<br>$[M]^-$	7.36	N/A	N/A	N/A	N/A	N/A	Sphingolipids (97%)	1.88	0.025	1.0	0.81	1.0
2<br>9	1070.9724<br>$[M]^-$	7.50	N/A	N/A	N/A	N/A	N/A	Sphingolipids (95%)	1.84	0.048	1.0	0.94	1.0

ESI Table 7: Two-way ANOVA of participant differences vs. minutes per timepoint, followed by a two-way ANOVA with Tukey's multiple comparison testing of the calculated minutes per timepoint:

Source of variation	% of total variation	P-value	P-value summary	Significant?
Participant	0.20	0.5917	ns	No
Minutes per timepoint	93	<0.0001	****	Yes

Two-way ANOVA	Mean Diff.	95.00% CI of diff.	Below threshold?	Summary	Adjusted P-value (Tukey's)
07:30 vs. 11:30	253	237 to 270	Yes	****	<0.0001
07:30 vs. 15:30	238	222 to 255	Yes	****	<0.0001
07:30 vs. 19:30	237	221 to 254	Yes	****	<0.0001
07:30 vs. 23:30	245	228 to 261	Yes	****	<0.0001
11:30 vs. 15:30	-15	-31 to 1.4	No	ns	0.0911
11:30 vs. 19:30	-16	-32 to 0.44	No	ns	0.0606
11:30 vs. 23:30	-8.7	-25 to 7.7	No	ns	0.5935
15:30 vs. 19:30	-0.97	-17 to 15	No	ns	0.9998
15:30 vs. 23:30	6.3	-10 to 23	No	ns	0.8250
19:30 vs. 23:30	7.3	-9.1 to 24	No	ns	0.7355

ESI Table 8: Timepoint effect evaluation using one-way ANOVA across timepoints and 24-hr rhythmicity evaluation by cosinor and Lomb-Scargle analyses across individuals. ANOVA testing was followed up with Tukey's multiple comparisons testing of the timepoint means. Text in red indicates a significant hit.

		ANOVA		Cosinor analysis		Lomb-Scargle analysis	
	Participant	P-value	Tukey's adj. P-value	P-value (acrophase)	FDR Q-value	P-value	FDR Q-value
Squalene	1	0.86	n.s.	0.098	0.47	0.18	0.43
	2	0.20	n.s.	0.03 (18:06)	0.17	0.16	0.38
	3	0.05*	n.s.*	0.54	0.93	0.43	0.72
	4	0.20	n.s.	0.05 (20:03)	0.22	0.16	0.31
	5	0.58	n.s.	0.58	0.96	0.43	0.80
	6	0.33	n.s.	0.58	1.00	0.47	0.88
	7	0.27	n.s.	0.87	1.00	0.78	0.92
	8	0.52	n.s.	0.50	0.84	0.39	0.66
Sapienic acid	1	0.60	n.s.	0.07	0.70	0.16	0.58
	2	0.85	n.s.	0.60	0.88	0.46	0.68

	3	0.64	n.s.	0.65	0.99	0.52	0.88
	4	0.07	n.s.	0.09	0.32	0.17	0.39
	5	0.53	n.s.	0.72	0.91	0.60	0.75
	6	0.23	n.s.	0.37	0.62	0.31	0.49
	7	0.84	n.s.	0.66	0.75	0.53	0.60
	8	0.67	n.s.	0.29	0.97	0.24	0.77
**Cholesterol sulfate**	1	0.81	n.s.	0.33	0.73	0.28	0.58
	2	0.84	n.s.	0.43	0.85	0.34	0.66
	3	0.90	n.s.	0.71	0.99	0.56	0.88
	4	0.37	n.s.	0.16	0.34	0.19	0.39
	5	0.91	n.s.	0.19	0.85	0.22	0.69
	6	0.74	n.s.	0.36	0.62	0.28	0.49
	7	0.44	n.s.	0.08	0.50	0.16	0.49
	8	0.95	n.s.	0.98	0.99	0.97	0.98

***Follow-up Tukey's multiple comparisons testing for Participant 3 ANOVA:**

One-way ANOVA	Mean diff.	95.00% CI of diff.	Below threshold?	Summary	Adjusted P-value (Tukey's)
08:00 vs. 12:00	0.04958	-0.1151 to 0.2143	No	n.s.	0.8913
08:00 vs. 16:00	-0.07830	-0.2430 to 0.08638	No	n.s.	0.6170
08:00 vs. 20:00	-0.07082	-0.2355 to 0.09387	No	n.s.	0.6984
08:00 vs. 24:00	0.04920	-0.1155 to 0.2139	No	n.s.	0.8939
12:00 vs. 16:00	-0.1279	-0.2832 to 0.02738	No	n.s.	0.1380
12:00 vs. 20:00	-0.1204	-0.2757 to 0.03487	No	n.s.	0.1780
12:00 vs. 24:00	-0.0003806	-0.1556 to 0.1549	No	n.s.	>0.9999
16:00 vs. 20:00	0.007489	-0.1478 to 0.1628	No	n.s.	0.9999
16:00 vs. 24:00	0.1275	-0.02776 to 0.2828	No	n.s.	0.1398
20:00 vs. 24:00	0.1200	-0.03525 to 0.2753	No	n.s.	0.1803

ESI Table 9: One-way ANOVA with Tukey's multiple comparison testing of participant differences vs. recovered squalene, sapienic acid, and cholesterol sulfate peak areas. Text in red indicates a significant hit.

Squalene:

One-way ANOVA	Mean diff.	95.00% CI of diff.	Below threshold?	Summary	Adjusted P-value (Tukey's)
1 vs. 2	-0.4657	-0.6232 to -0.3082	Yes	****	<0.0001
1 vs. 3	-0.3488	-0.5079 to -0.1896	Yes	****	<0.0001
1 vs. 4	-0.4004	-0.5579 to -0.2429	Yes	****	<0.0001
1 vs. 5	-0.6397	-0.7972 to -0.4822	Yes	****	<0.0001
1 vs. 6	-0.2922	-0.4497 to -0.1347	Yes	****	<0.0001
1 vs. 7	-0.4159	-0.5734 to -0.2584	Yes	****	<0.0001
1 vs. 8	-0.4461	-0.6036 to -0.2886	Yes	****	<0.0001
2 vs. 3	0.1169	-0.04224 to 0.2760	No	ns	0.3263
2 vs. 4	0.06523	-0.09227 to 0.2227	No	ns	0.9090
2 vs. 5	-0.1740	-0.3315 to -0.01655	Yes	*	0.0191
2 vs. 6	0.1734	0.01592 to 0.3309	Yes	*	0.0198
2 vs. 7	0.04971	-0.1078 to 0.2072	No	ns	0.9784
2 vs. 8	0.01954	-0.1380 to 0.1770	No	ns	>0.9999
3 vs. 4	-0.05167	-0.2108 to 0.1075	No	ns	0.9747
3 vs. 5	-0.2909	-0.4501 to -0.1318	Yes	****	<0.0001
3 vs. 6	0.05653	-0.1026 to 0.2157	No	ns	0.9585
3 vs. 7	-0.06718	-0.2263 to 0.09195	No	ns	0.9002
3 vs. 8	-0.09735	-0.2565 to 0.06178	No	ns	0.5698
4 vs. 5	-0.2393	-0.3968 to -0.08178	Yes	***	0.0002
4 vs. 6	0.1082	-0.04930 to 0.2657	No	ns	0.4152
4 vs. 7	-0.01551	-0.1730 to 0.1420	No	ns	>0.9999
4 vs. 8	-0.04568	-0.2032 to 0.1118	No	ns	0.9868
5 vs. 6	0.3475	0.1900 to 0.5050	Yes	****	<0.0001
5 vs. 7	0.2238	0.06626 to 0.3813	Yes	***	0.0006
5 vs. 8	0.1936	0.03609 to 0.3511	Yes	**	0.0053
6 vs. 7	-0.1237	-0.2812 to 0.03379	No	ns	0.2438
6 vs. 8	-0.1539	-0.3114 to 0.003621	No	ns	0.0608
7 vs. 8	-0.03017	-0.1877 to 0.1273	No	ns	0.9990

Sapienic acid:

One-way ANOVA	Mean diff.	95.00% CI of diff.	Below threshold?	Summary	Adjusted P-value (Tukey's)
1 vs. 2	-0.1613	-0.3981 to 0.07547	No	ns	0.4263
1 vs. 3	0.05771	-0.1842 to 0.2996	No	ns	0.9959
1 vs. 4	-0.08676	-0.3236 to 0.1500	No	ns	0.9511
1 vs. 5	-1.118	-1.355 to -0.8816	Yes	****	<0.0001
1 vs. 6	0.1406	-0.09618 to 0.3774	No	ns	0.6072
1 vs. 7	-0.03919	-0.2760 to 0.1976	No	ns	0.9996
1 vs. 8	-0.9564	-1.193 to -0.7196	Yes	****	<0.0001
2 vs. 3	0.2190	-0.02285 to 0.4609	No	ns	0.1075

2 vs. 4	0.07458	-0.1622 to 0.3114	No	ns	0.9787
2 vs. 5	-0.9570	-1.194 to -0.7202	Yes	****	<0.0001
2 vs. 6	0.3020	0.06516 to 0.5388	Yes	**	0.0032
2 vs. 7	0.1222	-0.1147 to 0.3590	No	ns	0.7611
2 vs. 8	-0.7950	-1.032 to -0.5582	Yes	****	<0.0001
3 vs. 4	-0.1445	-0.3864 to 0.09743	No	ns	0.6002
3 vs. 5	-1.176	-1.418 to -0.9342	Yes	****	<0.0001
3 vs. 6	0.08292	-0.1590 to 0.3248	No	ns	0.9658
3 vs. 7	-0.09689	-0.3388 to 0.1450	No	ns	0.9228
3 vs. 8	-1.014	-1.256 to -0.7722	Yes	****	<0.0001
4 vs. 5	-1.032	-1.268 to -0.7948	Yes	****	<0.0001
4 vs. 6	0.2274	-0.009422 to 0.4642	No	ns	0.0699
4 vs. 7	0.04757	-0.1892 to 0.2844	No	ns	0.9986
4 vs. 8	-0.8696	-1.106 to -0.6328	Yes	****	<0.0001
5 vs. 6	1.259	1.022 to 1.496	Yes	****	<0.0001
5 vs. 7	1.079	0.8424 to 1.316	Yes	****	<0.0001
5 vs. 8	0.1620	-0.07480 to 0.3988	No	ns	0.4207
6 vs. 7	-0.1798	-0.4166 to 0.05700	No	ns	0.2844
6 vs. 8	-1.097	-1.334 to -0.8602	Yes	****	<0.0001
7 vs. 8	-0.9172	-1.154 to -0.6804	Yes	****	<0.0001

Cholesterol sulfate:

One-way ANOVA	Mean diff.	95.00% CI of diff.	Below threshold?	Summary	Adjusted P-value (Tukey's)
1 vs. 2	-0.1410	-0.3480 to 0.06598	No	ns	0.4265
1 vs. 3	0.1896	-0.02179 to 0.4011	No	ns	0.1146
1 vs. 4	-0.4460	-0.6530 to -0.2391	Yes	****	<0.0001
1 vs. 5	-1.388	-1.595 to -1.181	Yes	****	<0.0001
1 vs. 6	0.03365	-0.1733 to 0.2406	No	ns	0.9997
1 vs. 7	0.06686	-0.1401 to 0.2738	No	ns	0.9754
1 vs. 8	-0.7702	-0.9772 to -0.5632	Yes	****	<0.0001
2 vs. 3	0.3306	0.1192 to 0.5421	Yes	****	<0.0001
2 vs. 4	-0.3050	-0.5120 to -0.09806	Yes	***	0.0003
2 vs. 5	-1.247	-1.454 to -1.040	Yes	****	<0.0001
2 vs. 6	0.1746	-0.03233 to 0.3816	No	ns	0.1675
2 vs. 7	0.2079	0.0008755 to 0.4148	Yes	*	0.0482
2 vs. 8	-0.6292	-0.8362 to -0.4222	Yes	****	<0.0001
3 vs. 4	-0.6357	-0.8471 to -0.4242	Yes	****	<0.0001
3 vs. 5	-1.577	-1.789 to -1.366	Yes	****	<0.0001
3 vs. 6	-0.1560	-0.3674 to 0.05543	No	ns	0.3206
3 vs. 7	-0.1228	-0.3342 to 0.08864	No	ns	0.6344
3 vs. 8	-0.9599	-1.171 to -0.7484	Yes	****	<0.0001
4 vs. 5	-0.9417	-1.149 to -0.7347	Yes	****	<0.0001

4 vs. 6	0.4797	0.2727 to 0.6867	Yes	****	<0.0001
4 vs. 7	0.5129	0.3059 to 0.7199	Yes	****	<0.0001
4 vs. 8	-0.3242	-0.5312 to -0.1172	Yes	****	<0.0001
5 vs. 6	1.421	1.214 to 1.628	Yes	****	<0.0001
5 vs. 7	1.455	1.248 to 1.662	Yes	****	<0.0001
5 vs. 8	0.6175	0.4105 to 0.8245	Yes	****	<0.0001
6 vs. 7	0.03321	-0.1738 to 0.2402	No	ns	0.9997
6 vs. 8	-0.8039	-1.011 to -0.5969	Yes	****	<0.0001
7 vs. 8	-0.8371	-1.044 to -0.6301	Yes	****	<0.0001

ESI Table 10: Level 2 annotations using GNPS and SIRIUS across data sets.

	Label	RT (min)	GNPS ID	GNPS cos score	GNPS shared peaks	SIRIUS ID	SIRIUS confidence score
RPLC+	V27	0.45	N/A	N/A	N/A	Panthenol	0.71
	V31	0.50	(2R,3R,4S,5S,6R)-2-[1,7-bis(4-hydroxyphenyl)heptan-3-yloxy]-6-[[(2S,3R,4R)-3,4-dihydroxy-4-(hydroxymethyl)oxolan-2-yl]oxymethyl]oxane-3,4,5-triol	0.87	15	N/A	N/A
	V41	0.51	N/A	N/A	N/A	2-{4-hydroxy-2,3-bis[(4-hydroxy-3,5-dimethoxyphenyl)methyl]butoxy}oxane-3,4,5-triol	0.79
	V125	0.75	N/A	N/A	N/A	Deet	0.72
	V130	0.75	Warfarin	0.99	12	4-hydroxy-3-(3-oxo-1-phenylbutyl)-2H-chromen-2-one	0.92
	V134	0.76	(Z)-2,6-dimethyl-7-(4-methyl-5-oxooxolan-2-yl)-3-[[3,4,5-trihydroxy-6-(hydroxymethyl)oxan-2-yl]oxymethyl]hept-5-enoic acid	0.76	6	N/A	N/A
	V135	0.76	CocamidopropylBetaine	0.96	6	2-[Dimethyl-[3-(2,2,3,3,4,4,5,5,6,6,7,7,8,8,9,9,10,10,11,11,12,12,12-tricosadeuteriododecanoylamino)propyl]azaniumyl]acetate	0.92
	V166	0.83	Lauryldiethanolamine	0.81	6	N/A	N/A
	V176	0.84	N/A	N/A	N/A	3-(Dimethylamino)propyl-(1-hydroxydodecylidene)azanium	0.86
	V237	1.01	N,N-Dimethyldodecylamine N-oxide	0.97	6	N/A	N/A
	V286	1.16	N/A	N/A	N/A	3-(Tetradecanoylamino)propyl(carboxymethyl)dimethylammonium	0.91

	V308	1.24	N-lauroylethanolamine	0.87	6	n-(2-hydroxyethyl)dodecanamide	0.78
	V313	1.24	Monolinolenin	0.89	10	N/A	N/A
	V345	1.33	N/A	N/A	N/A	Beiwutine	0.91
	V353	1.36	N/A	N/A	N/A	Myristamidopropylamine oxide	0.79
	V368	1.41	Lauric isopropanolamide	0.97	9	N/A	N/A
	V388	1.45	9-OxoOTrE	0.78	8	N/A	N/A
	V389	1.45	Benzyldodecyldimethylammonium	0.93	6	[Dideuterio-(2,3,4,5,6-pentadeuteriophenyl)methyl]-dodecyl-dimethylazanium	0.86
	V417	1.53	N/A	N/A	N/A	Phytosphingosine	0.72
	V425	1.55	N/A	N/A	N/A	2-aminoheptadecane-1,3-diol	0.88
	V478	1.72	LPC 18:2	0.90	12	N/A	N/A
	V490	1.78	D-erythro-C18-Sphingosine	0.83	8	Sphingoid	0.83
	V491	1.78	N-Tetracosenoyl-4-sphingenine	0.88	9	Sphingoid	0.88
	V493	1.79	9(10)-EpOME	0.92	10	N/A	N/A
	V528	1.87	Dibutyl adipate	0.93	8	N/A	N/A
	V534	1.89	N/A	N/A	N/A	Psychosine(1+)	0.81
	V559	1.93	Stearidonic acid	0.72	10	N/A	N/A
	V596	2.01	9-Oxo-10E,12Z-octadecadienoic acid	0.84	11	N/A	N/A
	V632	2.08	D-erythro-Sphinganine	0.96	11	Safingol	0.90
	V661	2.16	Oleic acid	0.74	7	N/A	N/A
	V662	2.16	Stearidonic acid	0.83	11	N/A	N/A

	V664	2.16	9-Oxo-10E,12Z-octadecadienoic acid	0.76	11	N/A	N/A
	V673	2.18	Myristoyl Ethanolamide|N-(2-hydroxyethyl)tetradecanamide	0.74	6	N/A	N/A
	V683	2.20	N/A	N/A	N/A	O-(1-O,2-O-Ditetradecyl-L-glycero-3-phospho)-2-aminoethanol	0.81
	V722	2.36	N/A	N/A	N/A	Hexadecanamide, N-[3-(dimethylamino)propyl]-	0.76
	V770	2.47	N/A	N/A	N/A	O-(1-O,2-O-Ditetradecyl-L-glycero-3-phospho)-2-aminoethanol	0.87
	V793	2.51	Palmitoylcarnitine	0.96	11	[(2R)-3-carboxy-2-(114C)hexadecanoyloxypropyl]-trimethylazanium	0.87
	V816	2.58	N/A	N/A	N/A	N-decyl-N'-[2-[(3-imino-2-oxotridecanoyl)amino]ethyl]oxamide	0.71
	V850	2.66	N/A	N/A	N/A	2-aminoicosane-1,3,4-triol	0.84
	V914	2.77	Palmitoyl ethanolamide	0.87	7	Palmitoylethanolamide	0.83
	V955	2.85	Linoleoyl ethanolamide	0.85	10	N-(2-hydroxyethyl)octadeca-9,12-dienamide	0.76
	V968	2.88	N/A	N/A	N/A	N-[3-(Dimethylamino)propyl]heptadecanamide	0.83
	V992	2.94	N/A	N/A	N/A	C20 Sphingosine	0.88
	V998	2.95	N/A	N/A	N/A	C20 Sphingosine	0.88
	V1025	3.00	3,6,9,12-Tetraoxatetracosan-1-ol	0.85	9	N/A	N/A
	V1027	3.00	N/A	N/A	N/A	Diethylene glycol monododecyl ether	0.74
	V1029	3.01	N/A	N/A	N/A	Triethyleneglycol Monolauryl Ether-d25	0.78
	V1030	3.01	N/A	N/A	N/A	Triethyleneglycol Monolauryl Ether-d25	0.77

	V1031	3.01	Diphenylphosphate	0.76	6	N/A	N/A
	V1118	3.19	Avobenzone	0.89	7	1-(4-tert-butylphenyl)-3-(4-methoxyphenyl)propane-1,3-dione	0.76
	V1122	3.19	3-Epilupeol	0.73	7	N/A	N/A
	V1143	3.27	Palmitoyl ethanolamide	0.97	9	Palmitoylethanolamide	0.80
	V1154	3.29	Octocrylene	0.90	9	2-ethylhexyl 2-cyano-3,3-diphenylprop-2-enoate	0.91
	V1177	3.32	N/A	N/A	N/A	Cer(t14:0/13:0)	0.83
	V1193	3.35	N/A	N/A	N/A	Octadecanamide, N-[3-(dimethylamino)propyl]-	0.91
	V1235	3.42	N/A	N/A	N/A	[3-Carboxylato-2-(stearoyloxy)propyl]trimethylaminium	0.84
	V1248	3.46	N-Oleoylethanolamine	0.98	11	N-(2-hydroxyethyl)octadec-9-enamide	0.72
	V1291	3.53	Sorbitane Monostearate - Polysorbate 60 in-source fragment	0.92	6	N/A	N/A
	V1341	3.62	N-[(1R)-2-Hydroxy-1-methylethyl-9Z-octadecenamide	0.93	8	N-(1-hydroxypropan-2-yl)octadec-9-enamide	0.86
	V1518	3.99	N/A	N/A	N/A	N-(2-hydroxyethyl)octadecanamide	0.84
	V1615	4.21	N/A	N/A	N/A	2-Aminotricosane-1,3-diol	0.87
	V1655	4.34	Bis(2-ethylhexyl) phthalate	0.97	7	Dehp	0.76
	V1751	4.57	Uvaol	0.73	7	N/A	N/A
	V1756	4.59	Glycerol Tri-n-octanoate	0.87	8	1,3-bis(octanoyloxy)propan-2-yl octanoate	0.70
	V1885	4.96	N/A	N/A	N/A	2-Aminoheptacosane-1,3-diol	0.84
	V1970	5.13	SM(d18:1/16:0)	0.93	6	N/A	N/A
	V2001	5.18	N/A	N/A	N/A	2,3-dihydroxypropyl hexacosanoate	0.86

	V2024	5.21	N/A	N/A	N/A	N-(1,3,4-trihydroxyoctadecan-2-yl)pentadecanamide	0.83
	V2034	5.23	TG(8:0/10:0/10:0)	0.74	9	N/A	N/A
	V2097	5.30	GalCer(d18:2/18:1)	0.94	21	N/A	N/A
	V2103	5.30	Tri-2-ethylhexyl trimellitate	0.75	6	N/A	N/A
	V2137	5.35	N/A	N/A	N/A	Gymnopilin A-10	1.00
	V2144	5.36	N/A	N/A	N/A	(9E,11E)-N-[(2S,3R)-1,3-dihydroxyoctadecan-2-yl]octadeca-9,11-dienamide	0.71
	V2207	5.44	AEG(o-14:1/14:1)	0.87	8	N/A	N/A
	V2209	5.44	N/A	N/A	N/A	6-O-[(2R,3R)-2-Decyl-3-hydroxytetradecanoyl]-alpha-D-glucopyranosyl 6-O-[(2R,3R)-2-decyl-3-hydroxytetradecanoyl]-alpha-D-glucopyranoside	0.74
	V2263	5.53	Glycerol 1-myristate	0.90	8	N/A	N/A
	V2271	5.54	N-[1,3-dihydroxyoctadec-4-en-2-yl]hexadecanamide	0.84	10	N/A	N/A
	V2272	5.54	Ceramide (18:1/16:0)	0.86	10	N/A	N/A
	V2302	5.58	alpha-tochopheryl acetate	0.97	15	2,5,7,8-tetramethyl-2-(4,8,12-trimethyltridecyl)-3,4-dihydro-2H-1-benzopyran-6-yl acetate	0.96
	V2304	5.58	alpha-tochopheryl acetate	0.97	13	2,5,7,8-tetramethyl-2-(4,8,12-trimethyltridecyl)-3,4-dihydro-2H-1-benzopyran-6-yl acetate	0.97
	V2305	5.58	Monopalmitolein (9c)	0.88	9	N/A	N/A
	V2331	5.62	DG(16:1/18:2/0:0)	0.72	16	N/A	N/A
	V2344	5.65	18:1(2S-OH) Ceramide	0.85	13	N/A	N/A
	V2386	5.71	N/A	N/A	N/A	[1-[(Z)-hexadec-9-enoyl]oxy-3-hydroxypropan-2-yl] (Z)-heptadec-9-enoate	0.72

	V2422	5.77	AEG(o-14:1/16:1)	0.91	11	N/A	N/A
	V2444	5.79	Glycerol 1-myristate	0.88	8	N/A	N/A
	V2460	5.82	Ceramide (d18:0/17:0)	0.79	15	N/A	N/A
	V2477	5.85	N/A	N/A	N/A	N,N'-[Iminobis(2,1-ethanediylimino-2,1-ethanediyl)]bis(tetradecanamide)	1.00
	V2481	5.86	Monopalmitolein (9c)	0.71	8	N/A	0.10
	V2486	5.86	Cer(d18:2/20:1)	0.76	13	N/A	N/A
	V2493	5.87	N-[1,3-dihydroxyoctadec-4-en-2-yl]octadecanamide	0.81	9	N/A	N/A
	V2510	5.88	Monoelaidin	0.93	10	N/A	N/A
	V2531	5.91	GalCer(d18:2/22:1)	0.85	18	N/A	N/A
	V2578	5.98	Cer(d18:0/18:0)	0.77	9	N/A	N/A
	V2622	6.07	AEG(o-16:3/15:0)	0.78	11	N/A	N/A
	V2637	6.10	AEG(o-16:2/16:0)	0.79	8	N/A	N/A
	V2640	6.10	N/A	N/A	N/A	2-[(Z)-hexadec-9-enoyl]oxypropyl (Z)-hexadec-9-enoate	0.74
	V2654	6.13	1-Palmitoyl-2-oleoyl-sn-glycerol	0.89	10	N/A	N/A
	V2665	6.15	Monoelaidin	0.89	10	N/A	N/A
	V2670	6.15	AEG(o-18:3/18:1)	0.76	10	N/A	N/A
	V2684	6.17	N/A	N/A	N/A	(14Z,16Z)-N-(1,3-dihydroxynonadecan-2-yl)docosa-14,16-dienamide	0.70
	V2704	6.21	N/A	N/A	N/A	3,4-dihydroxy-N-(1,3,6-trihydroxypentadec-4-en-2-yl)pentacosanamide	0.74
	V2768	6.31	N/A	N/A	N/A	1-O-[2,3-di(tetradecanoyloxy)propyl] 14-O-ethenyl	0.99

						tetradecanedioate	
	V2780	6.33	Cer(d18:0/20:0)	0.74	13	N/A	N/A
	V2802	6.36	Squalene	0.82	16	N/A	N/A
	V2894	6.48	Sclareol	0.79	10	N/A	N/A
	V2978	6.59	N/A	N/A	N/A	Myristic acid, ester with oxybispropanediol (3:1)	0.95
	V3026	6.66	Cer(d18:0/22:0)	0.77	13	N/A	N/A
	V3037	6.67	N/A	N/A	N/A	2,3-Di(tetradec-9-enoyloxy)propyl tetradec-9-enoate	0.71
	V3053	6.70	N/A	N/A	N/A	1-O-Lauroyl-2-O-myristoyl-3-O-(9,10-epoxy-1-oxooctadecane-1-yl)-L-glycerol	0.75
	V3060	6.71	Cholesta-4,6-dien-3-one	0.82	9	9a,11a-dimethyl-1-(6-methylheptan-2-yl)-1H,2H,3H,3aH,3bH,7H,8H,9H,9aH,9bH,10H,11H,11aH-cyclopenta[a]phenanthren-7-one	0.77
	V3062	6.71	Cer(d18:1/24:1)	0.71	9	N/A	N/A
	V3067	6.72	N/A	N/A	N/A	[2-[(9Z,12Z)-heptadeca-9,12-dienoyl]oxy-3-[(E)-17-oxoheptadec-9-enoyl]oxypropyl] octadecanoate	0.92
	V3071	6.72	N/A	N/A	N/A	5-(3,7,11,15,19,23,27,31,35,39-decamethyltetraconta-2,6,10,14,18,22,26,30,34,38-decaen-1-yl)-2,3-dimethylcyclohexa-2,5-diene-1,4-dione	1.00
	V3076	6.74	N/A	N/A	N/A	(3-Hydroperoxy-2-octadecoxypropyl) octadecanoate	0.85
	V3134	6.83	TAG (12:0/12:0/14:0)	0.75	18	N/A	N/A
	V3182	6.89	N/A	N/A	N/A	4-Piperidinecarboxylic acid, 1-(4-(4-morpholinylcarbonyl)-2,3,5,6-tetraiodobenzoyl)-	0.98
	V3247	6.97	Cer(d18:0/24:0)	0.75	13	N/A	N/A

	V3275	7.02	Cholesta-4,6-dien-3-one	0.82	9	N/A	N/A
	V3282	7.03	N/A	N/A	N/A	[2-[(9Z,12Z)-heptadeca-9,12-dienoyl]oxy-3-[(E)-17-oxoheptadec-9-enoyl]oxypropyl] octadecanoate	0.97
	V3297	7.05	N/A	N/A	N/A	[2-[(9Z,12Z)-heptadeca-9,12-dienoyl]oxy-3-[(E)-17-oxoheptadec-9-enoyl]oxypropyl] octadecanoate	0.97
	V3343	7.13	TAG (12:0/14:0/16:0)	0.81	19	N/A	N/A
	V3351	7.14	NCGC00347805-02!(2E,6E)-3,7,11,15,19,23,27,31,35-nonamethylhexatriaconta-2,6,34-triene-1,10,11,15,19,23,27,31-octol	0.75	6	N/A	N/A
	V3366	7.16	N/A	N/A	N/A	5-(3,7,11,15,19,23,27,31,35,39-decamethyltetraconta-2,6,10,14,18,22,26,30,34,38-decaen-1-yl)-2,3-dimethylcyclohexa-2,5-diene-1,4-dione	0.94
	V3381	7.16	N/A	N/A	N/A	Oxo-di(tricosan-2-yloxy)phosphanium	0.75
	V3395	7.17	N/A	N/A	N/A	1-O-Palmitoyl-2-O-oleoyl-3-O-(9,10-epoxy-1-oxooctadecane-1-yl)-L-glycerol	0.82
	V3406	7.20	N/A	N/A	N/A	1-O,2-O-Dioleoyl-3-O-[(E)-1-oxo-12,13-epoxy-9-octadecene-1-yl]-L-glycerol	0.97
	V3410	7.21	N/A	N/A	N/A	2,3-dihydroxypropyl hexacosanoate	0.87
	V3422	7.23	N/A	N/A	N/A	1-O-Myristoyl-2-O-oleoyl-3-O-(9,10-epoxy-1-oxooctadecane-1-yl)-L-glycerol	0.81
	V3431	7.24	Desmosterol	0.79	12	N/A	N/A
	V3491	7.32	12(13)-Epoxy-9Z-octadecenoic acid	0.72	9	N/A	N/A
	V3527	7.39	Ubidecarenone	0.93	12	N/A	N/A
	V3528	7.39	Ubidecarenone	0.94	12	N/A	N/A

	V3536	7.41	N/A	N/A	N/A	1,2-Propanediol dibehenate	0.73
	V3541	7.41	TG(13:0/13:0/16:0)	0.76	17	N/A	N/A
	V3568	7.44	AEG(o-14:1/14:1)	0.88	8	N/A	N/A
	V3608	7.49	AEG(o-14:1/16:2)	0.94	13	N/A	N/A
	V3611	7.49	TG(14:1/16:0/16:1)	0.71	18	N/A	N/A
	V3626	7.52	TG(12:0/15:0/16:0)	0.73	14	N/A	N/A
	V3631	7.53	AEG(o-16:3/16:1)	0.83	11	N/A	N/A
	V3644	7.55	Cer(d20:0/26:0)	0.80	16	N/A	N/A
	V3657	7.57	TG(14:0/15:1/16:0)	0.71	18	N/A	N/A
	V3682	7.61	AEG(o-16:3/15:0)	0.84	15	N/A	N/A
	V3686	7.62	TG(15:0/16:1/16:1)	0.71	20	N/A	N/A
	V3714	7.67	TAG (14:0/14:0/16:0)	0.75	17	N/A	N/A
	V3715	7.68	AEG(o-14:1/14:1)	0.87	7	N/A	N/A
	V3734	7.71	AEG(o-14:1/16:1)	0.95	11	N/A	N/A
	V3741	7.71	TG(14:0/16:0/16:1)	0.72	19	N/A	N/A
	V3749	7.72	AEG(o-14:1/16:2)	0.75	9	N/A	N/A
	V3766	7.75	AEG(o-16:3/16:0)	0.90	13	N/A	N/A
	V3771	7.75	AEG(o-16:3/16:1)	0.74	11	N/A	N/A
	V3784	7.77	AEG(o-16:3/18:1)	0.92	13	N/A	N/A
	V3831	7.84	AEG(o-16:3/15:0)	0.83	15	N/A	N/A

	V3872	7.89	N/A	N/A	N/A	1,2,3-tri-(9Z-heptadecenoyl)-glycerol	0.72
	V3890	7.92	AEG(o-18:3/16:1)	0.91	13	N/A	N/A
	V3893	7.92	TAG (14:0/16:0/16:0)/TAG (14:0/14:0/18:0)	0.72	17	N/A	N/A
	V3895	7.92	AEG(o-14:1/16:1)	0.96	11	N/A	N/A
	V3913	7.96	AEG(o-16:3/16:0)	0.82	12	N/A	N/A
	V3915	7.96	N/A	N/A	N/A	[3-[(Z)-heptadec-9-enoyl]oxy-2-[(9Z,12Z)-nonadeca-9,12-dienoyl]oxypropyl] (9Z,12Z)-nonadeca-9,12-dienoate	0.71
	V3927	7.97	AEG(o-16:2/16:0)	0.86	9	N/A	N/A
	V3938	7.98	TG(16:0/16:1/18:1)	0.73	18	N/A	N/A
	V3945	7.98	1-Palmitoyl-2-oleoyl-sn-glycerol	0.79	9	N/A	N/A
	V4009	8.07	N/A	N/A	N/A	[2-[(9Z,12Z)-nonadeca-9,12-dienoyl]oxy-3-[(Z)-octadec-9-enoyl]oxypropyl] (9Z,12Z)-nonadeca-9,12-dienoate	0.71
	V4021	8.05	1-Palmitoyl-2-oleoyl-sn-glycerol	0.90	10	N/A	N/A
	V4029	8.11	TG(17:1/18:1/18:1)	0.73	15	N/A	N/A
	V4031	8.11	N/A	N/A	N/A	[10,13-dimethyl-17-[4-(2-methylcyclopropyl)but-3-en-2-yl]-2,3,4,5,6,7,8,9,11,12,14,15,16,17-tetradecahydro-1H-cyclopenta[a]phenanthren-3-yl] 4,8,12-trimethyltridecanoate	0.76
	V4034	8.11	Cholesterol	0.95	17	N/A	N/A
	V4038	8.11	N/A	N/A	N/A	TAG (17:1/18:1/18:1)	0.76
	V4073	8.17	AEG(o-18:3/18:1)	0.88	11	N/A	N/A
	V4080	8.18	TAG (16:0/16:0/18:1)	0.71	17	N/A	N/A

	V4097	8.20	Cholesterol	0.95	17	N/A	N/A
	V4128	8.24	N/A	N/A	N/A	2-methyl-3-[(3R,27S,31R)-3,7,11,15,19,23,27,31,35-nonamethylhexatriacontyl]naphthalene-1,4-diol	1.00
	V4205	8.35	Cholesterol	0.93	15	N/A	N/A
	V4252	8.41	TG(18:1/18:1/20:1)	0.74	21	N/A	N/A
	V4308	8.50	N/A	N/A	N/A	[3-[(Z)-hexadec-9-enoyl]oxy-2,2-bis[[(Z)-hexadec-9-enoyl]oxymethyl]propyl] (Z)-hexadec-9-enoate	0.72
	V4413	8.69	N/A	N/A	N/A	3,7,11,15,19,23,27,31,35,39,43,47,51-tridecamethyldopentaconta-2,6,10,14,18,22,26,30,34,38,42,46,50-tridecaen-1-ol	0.97
	V4460	8.79	N/A	N/A	N/A	Glycerol tri-(13-docosynoate)	0.77
	V4546	8.96	N/A	N/A	N/A	(2-Nonadec-1-en-2-yloxy-3-octadecanoyloxypropyl) octadecanoate	0.79
	V4572	9.02	Cholesterol	0.91	16	N/A	N/A
	V4595	9.07	N/A	N/A	N/A	Nonapentaconta-2,57-dienedioic acid	0.70
	V4642	9.20	Cholesterol	0.91	16	N/A	N/A
	V4665	9.29	N/A	N/A	N/A	11-[17-(6-carboxy-2,3,4-trihydroxyphenoxy)-3,4,5,18,19-pentahydroxy-8,14-dioxo-11-(3,4,5-trihydroxybenzoyloxy)-9,13-dioxatricyclononadeca-1(15),2,4,6,16,18-hexaen-10-yl]-3,4,5,16,17,18-hexahydroxy-8,13-dioxo-9,12-dioxatricyclo[12.4.0.0Â²,â�·]octadeca-1(14),2,4,6,15,17-hexaene-10-carboxylic acid	1.00
	V4692	9.39	Cholesterol	0.90	14	N/A	N/A
**RP**	V10	0.43	Catechin	0.91	12	Catechin	0.75

	V15	0.43	(2R,3R,4S,5S,6R)-2-[1,7-bis(4-hydroxyphenyl)heptan-3-yloxy]-6-[[(2S,3R,4R)-3,4-dihydroxy-4-(hydroxymethyl)oxolan-2-yl]oxymethyl]oxane-3,4,5-triol	0.86	15	N/A	N/A
	V47	0.51	1,7-bis(4-hydroxyphenyl)heptane-3,5-diol	0.84	8	N/A	N/A
	V75	0.53	(2R,3S,4S,5R,6R)-2-[[(2S,3R,4R)-3,4-dihydroxy-4-(hydroxymethyl)oxolan-2-yl]oxymethyl]-6-[4-(4-hydroxyphenyl)butan-2-yloxy]oxane-3,4,5-triol	0.90	13	N/A	N/A
	V117	0.62	Platyphyllenone	0.96	7	N/A	N/A
	V310	1.02	Azelaic acid	0.92	6	N/A	N/A
	V378	1.20	N/A	N/A	N/A	(2R)-2-(hexadecanoylamino)-3-hydroxypropanoic acid	0.75
	V408	1.21	N/A	N/A	N/A	1-Hexadecanoyl-2-azeloyl-sn-glycero-3-phospho-(1'-myo-inositol)	0.98
	V445	1.32	N/A	N/A	N/A	(2S)-2-(hexadecanoylamino)-3-hydroxybutanoic acid	0.90
	V490	1.48	1-Octadecanoyl-sn-glycero-3-phospho-(1'-sn-glycerol)	0.93	7	N/A	N/A
	V568	1.62	Azelaic acid	0.93	7	N/A	N/A
	V630	1.70	PC 34:2	0.82	7	N/A	N/A
	V658	1.84	N/A	N/A	N/A	9-(5-Carboxypentanoyloxy)-10-(6-oxohexanoyloxy)octadecanoic acid	0.83
	V774	2.17	PC 30:0	0.79	9	N/A	N/A
	V832	2.27	N-Oleoyl-Phenylalanine	0.74	7	N/A	N/A
	V846	2.29	Phytoceramide C2	0.72	10	N/A	N/A
'	V867	2.39	Azelaic acid	0.94	6	N/A	N/A

	V1463	3.65	N/A	N/A	N/A	2-(2-Octadecoxyethoxy)ethyl hydrogen sulfate	0.75
	V1617	4.08	N/A	N/A	N/A	3-(16-ethenyl-11-ethyl-4-hydroxy-12,17,21,26-tetramethyl-7,23,24,25-tetrazahexacyclo[18.2.1.15,8.110,13.115,18.02,6]hexacosan-22-yl)-N-[2,4,5-trihydroxy-6-(hydroxymethyl)oxan-3-yl]propanamide	0.85
	V1718	4.41	N/A	N/A	N/A	2-hydroxy-N-[(E,2S,3S)-1,3,4,5-tetrahydroxyoctadec-4-en-2-yl]hexadecanamide	0.97
	V1725	4.43	N/A	N/A	N/A	2-amino-9-[2-oxo-1-(4-oxohexadecoxy)tetradecyl]-7,8-dihydro-1H-purin-6-one	0.91
	V1754	4.53	N/A	N/A	N/A	[1-oxo-1-[[(2S,3S,4R)-1,3,4-trihydroxyoctadecan-2-yl]amino]decan-2-yl] acetate	0.97
	V1943	5.03	N/A	N/A	N/A	3-[4-[3-[3-[4-(4-cycloheptylbut-1-en-2-ylamino)phenyl]propanoyl]anilino]-4-oxobutyl]-N-[4-(3-cycloheptylpropanoylamino)phenyl]benzamide	0.83
	V1958	5.07	N/A	N/A	N/A	2-hydroxy-N-[(E,2S,3S)-1,3,4,5-tetrahydroxyoctadec-4-en-2-yl]hexadecanamide	0.88
	V1959	5.07	N/A	N/A	N/A	2-hydroxy-N-[(E,2S,3S)-1,3,4,5-tetrahydroxyoctadec-4-en-2-yl]hexadecanamide	0.89
	V2018	5.23	N/A	N/A	N/A	octyl-[(1S,2R,4R,5S)-2,3,4-trihydroxy-5,6-bis[[hydroxy(octyl)boranyl]oxy]cyclohexyl]oxyborinic acid	0.72
	V2041	5.28	N-[1,3-dihydroxyoctadec-4-en-2-yl]hexadecanamide	0.92	11	N/A	N/A
	V2072	5.37	N-(1.3-dihydroxyoctadeca-4.14-dien-2-yl)palmitamide	0.81	7	N/A	N/A
	V2133	5.54	N-[1.3-dihydroxyoctadec-4-en-2-yl]docosanamide	0.92	9	N/A	N/A
	V2164	5.61	N/A	N/A	N/A	Tert-butyl 2-[2-(2-bicyclo[2.2.1]hept-5-enyl)ethyl]-7-[7-[2-[2-(2-bicyclo[2.2.1]hept-5-enyl)ethyl]-5-hydroxy-5-	0.85

						methylhexanoyl]oxy-6,6,7-trimethyloctanoyl]oxy-6,6,7-trimethyloctanoate	
	V2346	6.06	N/A	N/A	N/A	(2R)-N-(1,2-dihydroxyethyl)-2-hydroxy-N-[(E,3S,4R)-1,3,4-trihydroxyhexacos-8-en-2-yl]heptadecanamide	0.75
	V2357	6.07	N-[1.3-dihydroxyoctadec-4-en-2-yl]octadecanamide	0.75	12	N/A	N/A
	V2429	6.26	N/A	N/A	N/A	2,3-dihydroxy-N-(1,3,4-trihydroxyicosan-2-yl)hexacosanamide	0.98
	V2474	6.33	N/A	N/A	N/A	(4R,5R)-5-heptadecyl-2,2-dimethyl-N-[(2S,3S,4R)-1,3,4-trihydroxydocosan-2-yl]-1,3-dioxolane-4-carboxamide	0.95
	V2481	6.34	N-[1.3-dihydroxyoctadec-4-en-2-yl]docosanamide	0.80	11	N/A	N/A
	V2488	6.38	N/A	N/A	N/A	N-[1-[(4,5-dihydroxy-7-oxabicyclo[4.1.0]heptan-3-yl)oxy]-3-hydroxyoctadecan-2-yl]hexacosanamide	0.76
	V2707	6.90	N/A	N/A	N/A	Cer(t18:1(6OH)/32:0(32OH))	0.71
	V2725	6.95	N/A	N/A	N/A	Cer(t18:1(6OH)/31:0(31OH))	0.96
	V2773	7.13	N/A	N/A	N/A	Cer(t18:1(6OH)/34:0(34OH))	0.96
	V2788	7.19	N/A	N/A	N/A	Cer(t18:1(6OH)/33:0(33OH))	0.96
	V2814	7.30	N/A	N/A	N/A	Cer(t18:1(6OH)/34:0(34OH))	0.96
	V2818	7.35	N/A	N/A	N/A	Cer(t18:1(6OH)/36:0(36OH))	0.95
	V2866	7.68	N/A	N/A	N/A	Ceramide NS dilaurate	0.89

Figures:

ESI Figure 1: Diagram showing the sampling area used for the study.

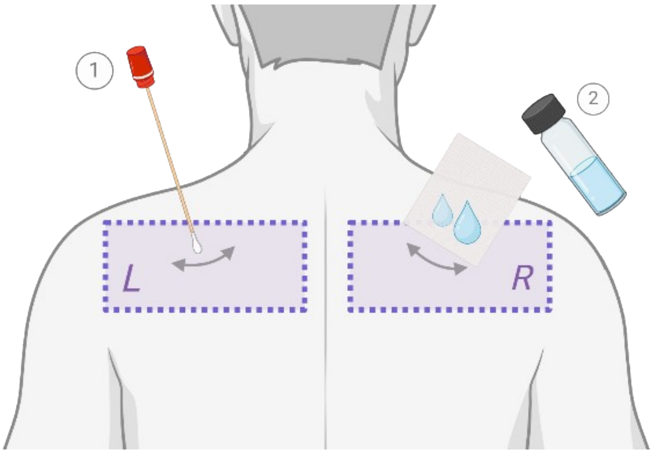

ESI Figure 2: PCA analysis conducted on samples with QC samples included, showing tight clustering about the origin, indicating good instrument performance and repeatability of sample brackets.

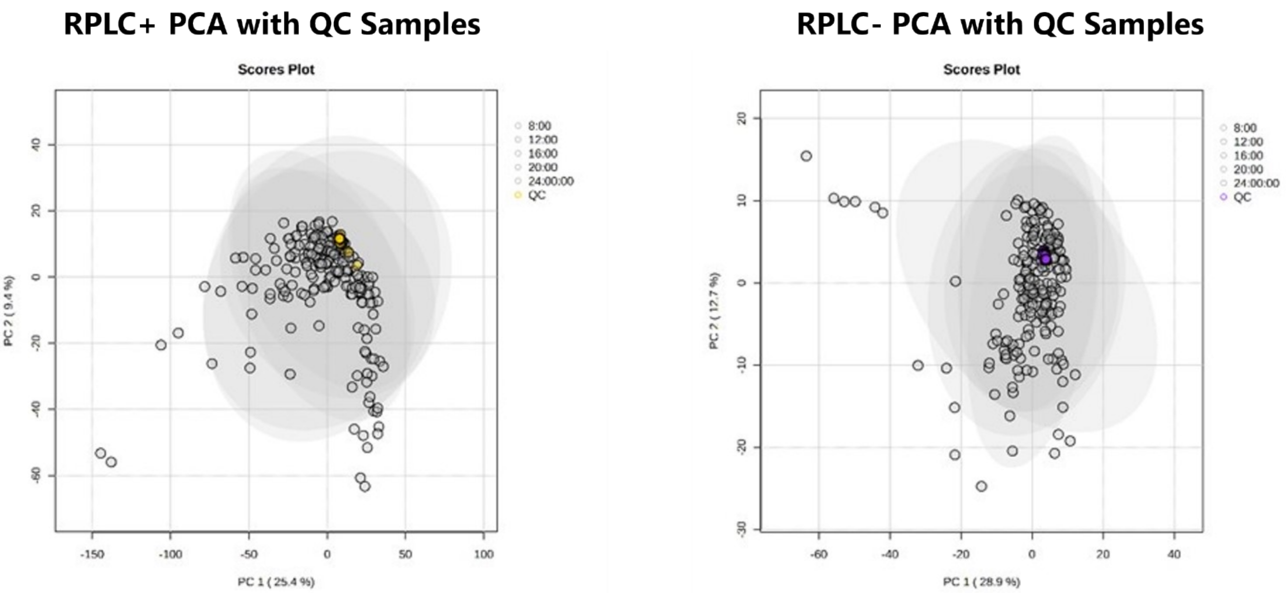

ESI Figure 3: PLS-DA model cross validation (5-fold) for the first 5 components, showing low accuracy (< 20%) and negative values for Q2 in all components, showing that the model is not at all predictive or is overfitted.

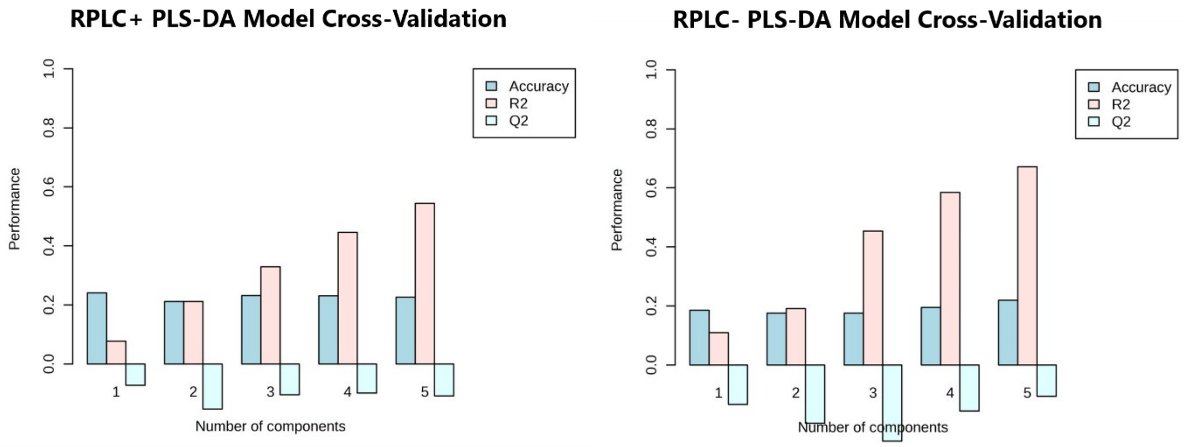

ESI Figure 4: Example RPLC ESI+ and ESI- TIC chromatograms (red = QC, black = swab blank).

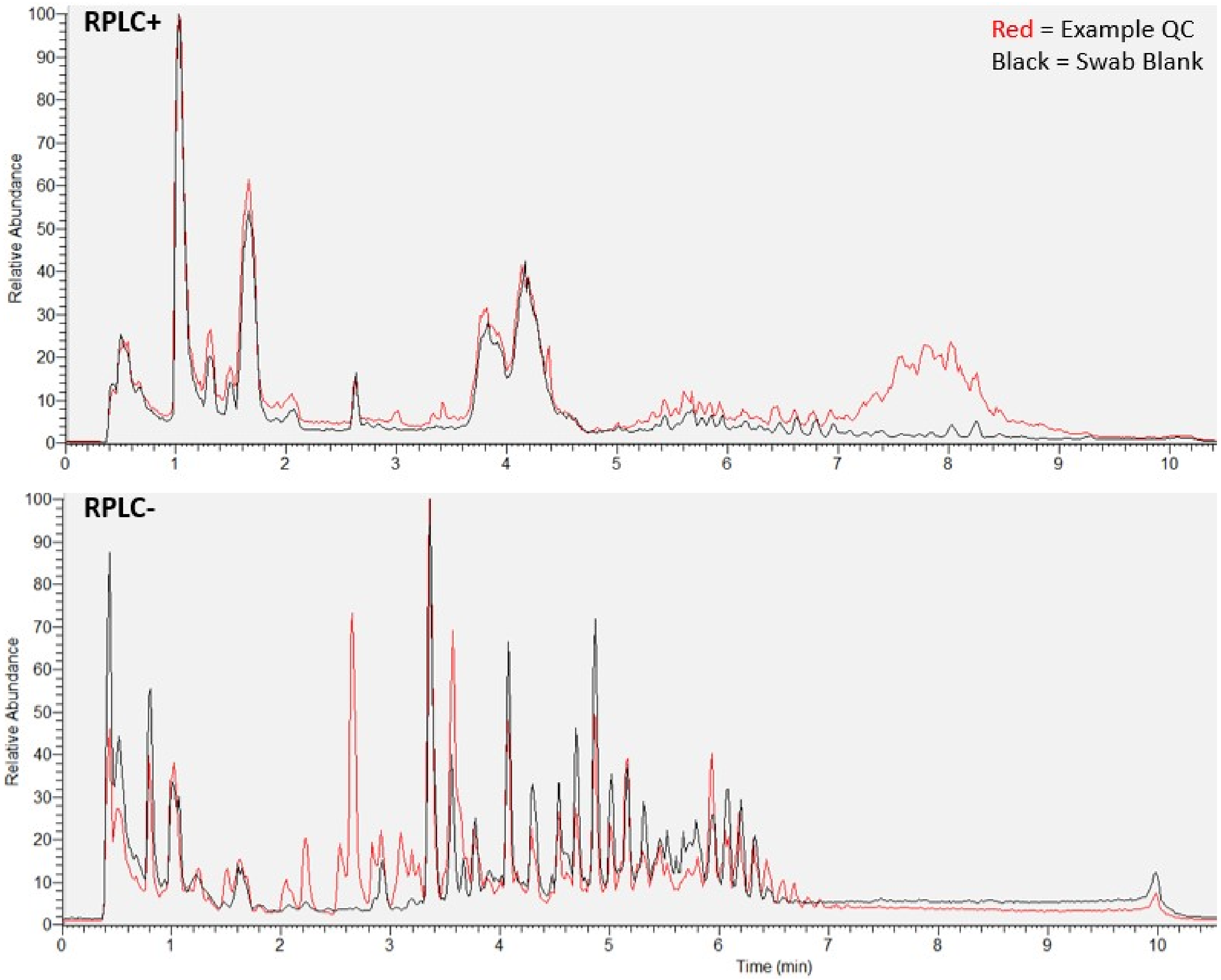

ESI Figure 5: TIC chromatograms of example cosmetic contaminant peaks (purple = QC, grey = swab blank, highlighted peak = EIC of contaminant *m/z* from QC sample). These chromatograms show that contaminants are not on present on the untreated swabs and must come from the sample.

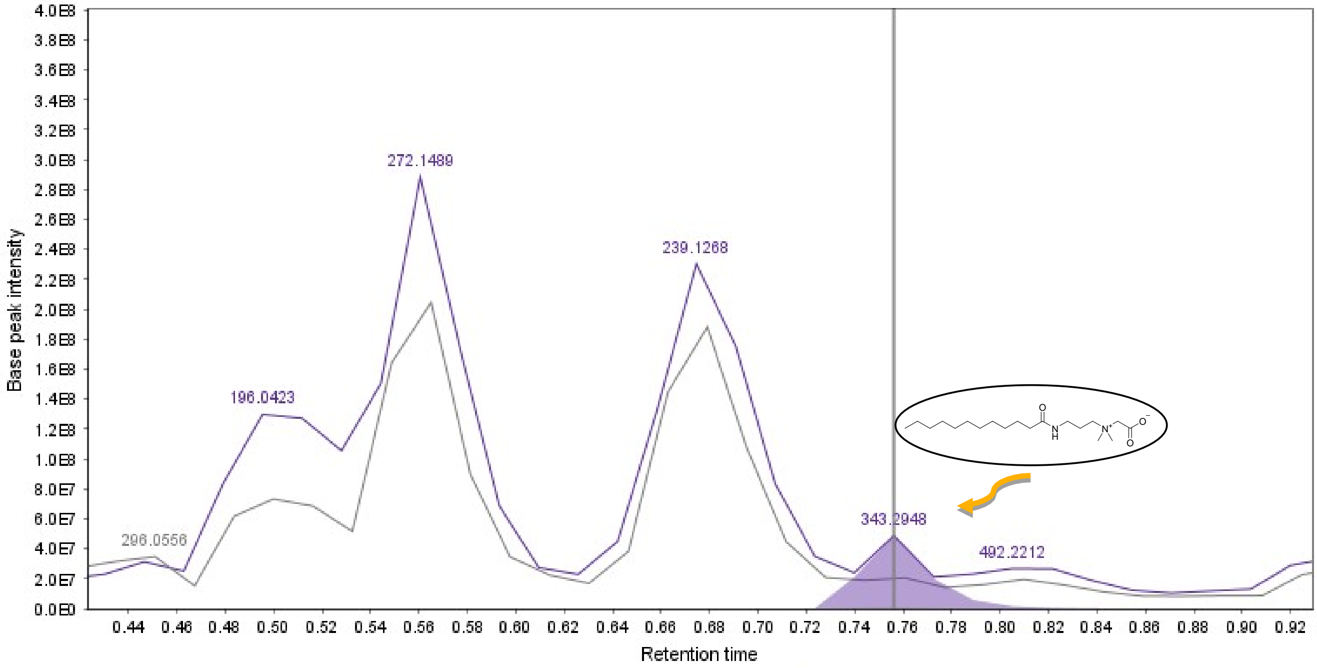

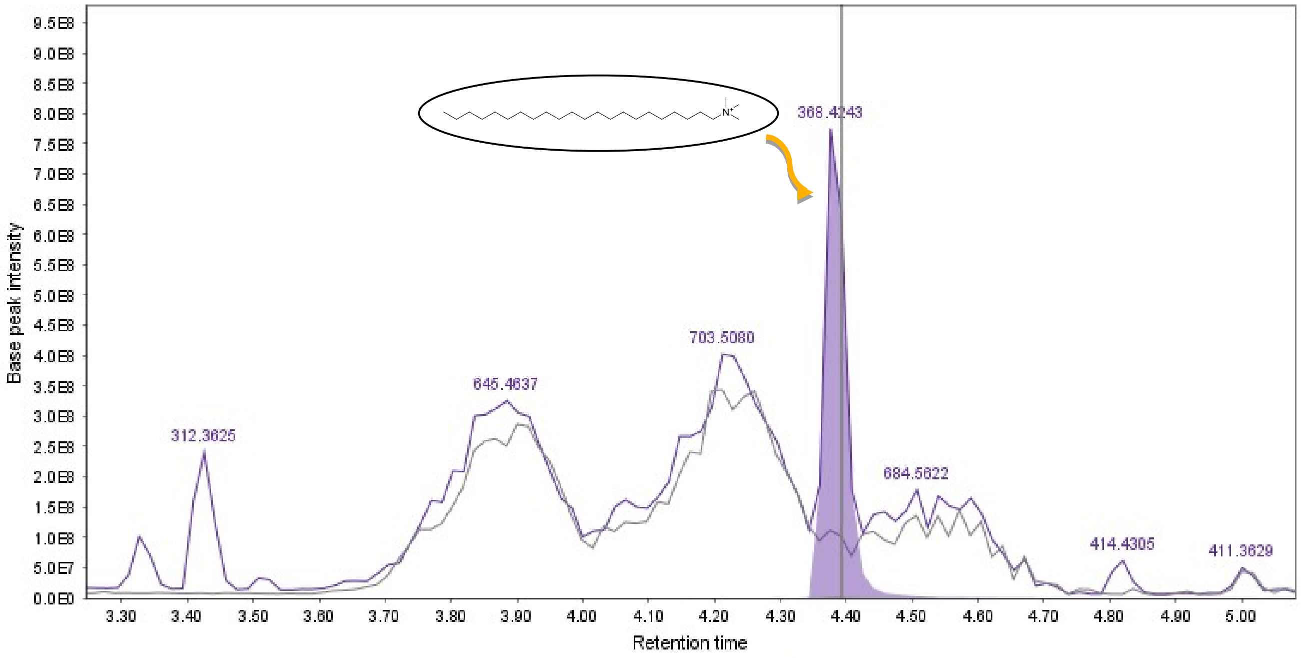

Supplement: AN-150-D5AN00665A-s001 [file AN-150-D5AN00665A-s001.pdf]
